# In vitro co-culture systems of hepatic and intestinal cells for cellular pharmacokinetic and pharmacodynamic studies of capecitabine against colorectal cancer

**DOI:** 10.1186/s12935-023-02853-6

**Published:** 2023-01-31

**Authors:** Chun Ge, Xintong Huang, Sujie Zhang, Man Yuan, Zhaoyi Tan, Chen Xu, Qiong Jie, Jingjing Zhang, Jianjun Zou, Yubing Zhu, Dong Feng, Yue Zhang, Jiye Aa

**Affiliations:** 1 Department of Pharmacy, Nanjing First Hospital, Nanjing Medical University, Nanjing, 210006 China; 2 Department of Clinical Pharmacology Lab, Nanjing First Hospital, Nanjing Medical University, Nanjing, 210006 China; 3 Department of Clinical Pharmacy, School of Basic Medicine & Clinical Pharmacy, China Pharmaceutical University, Nanjing, 211198 China; 4 Key Laboratory of Drug Metabolism and Pharmacokinetics, State Key Laboratory of Natural Medicines, China Pharmaceutical University, Nanjing, 210009 China; 5 Nanjing Southern Pharmaceutical Technology Co., Ltd., Nanjing, 211100 China

**Keywords:** Cellular pharmacokinetics, Cellular pharmacodynamics, Capecitabine, Co-culture, Prodrug

## Abstract

**Background:**

As a prodrug of 5-fluorouracil (5-FU), orally administrated capecitabine (CAP) undergoes preliminary conversion into active metabolites in the liver and then releases 5-FU in the gut to exert the anti-tumor activity. Since metabolic changes of CAP play a key role in its activation, a single kind of intestinal or hepatic cell can never be used in vitro to evaluate the pharmacokinetics (PK) and pharmacodynamics (PD) nature. Hence, we aimed to establish a novel in vitro system to effectively assess the PK and PD of these kinds of prodrugs.

**Methods:**

Co-culture cellular models were established by simultaneously using colorectal cancer (CRC) and hepatocarcinoma cell lines in one system. Cell Counting Kit-8 (CCK-8) and flow cytometric analysis were used to evaluate cell viability and apoptosis, respectively. Apoptosis-related protein expression levels were measured using western blot analysis. A selective liquid chromatography-tandem mass spectrometry (LC–MS/MS) method was developed for cellular PK in co-culture models.

**Results:**

CAP had little anti-proliferative effect on the five monolayer CRC cell lines (SW480, LoVo, HCT-8, HCT-116 and SW620) or the hepatocarcinoma cell line (HepG2). However, CAP exerted marked anti-tumor activities on each of the CRC cell lines in the co-culture models containing both CRC and hepatocarcinoma cell lines, although its effect on the five CRC cell lines varied. Moreover, after pre-incubation of CAP with HepG2 cells, the culture media containing the active metabolites of CAP also showed an anti-tumor effect on the five CRC cell lines, indicating the crucial role of hepatic cells in the activation of CAP.

**Conclusion:**

The simple and cost‑effective co-culture models with both CRC and hepatocarcinoma cells could mimic the in vivo process of a prodrug dependent on metabolic conversion to active metabolites in the liver, providing a valuable strategy for evaluating the PK and PD characteristics of CAP-like prodrugs in vitro at the early stage of drug development.

**Supplementary Information:**

The online version contains supplementary material available at 10.1186/s12935-023-02853-6.

## Background

Colorectal cancer (CRC) is the most common gastrointestinal tumor and the second leading cause of cancer-related deaths worldwide [1]. Despite recent progress in the management of CRC, the 5 year survival and disease-free survival remain far from satisfactory [2]. Notably, nearly one-third of the patients diagnosed with CRC develop tumor metastasis that involves multiple organs, which presents a great challenge for detection and treatment [3, 4]. However, since many patients with CRC are unsuitable for surgical treatment, or have missed the best time for surgery, chemotherapy remains the conventional treatment, although the therapeutic benefit is limited by toxicity to normal cells and resistance to multi-drugs [5, 6].

**Fig. 1:**
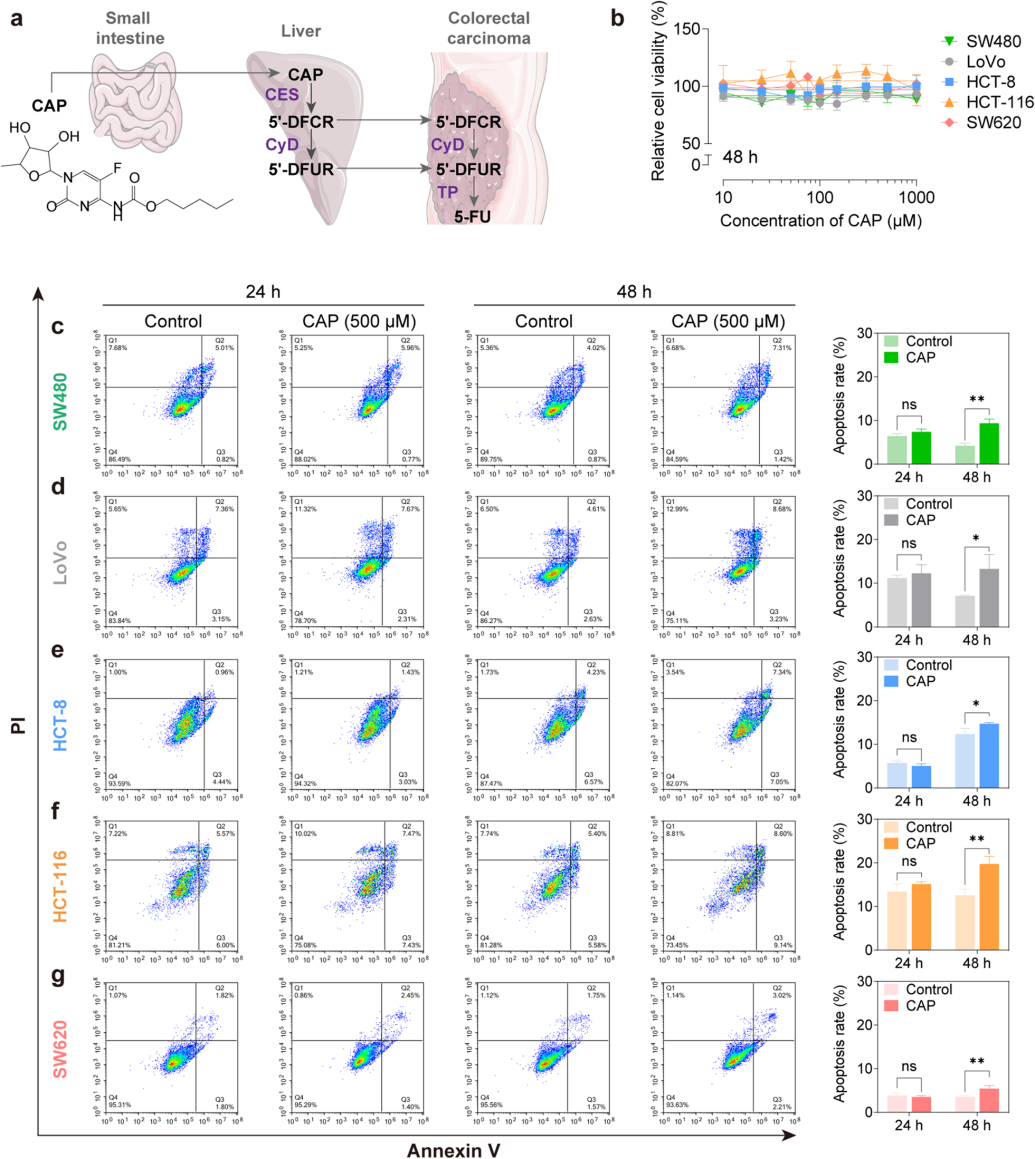
CAP exhibited little inhibitory effect on CRC cells under the condition of inactivation by the liver. **a** The structure of CAP and metabolic conversion of CAP to 5-FU. **b** Relative cell viability of CRC cells measured by CCK-8 assay after treated with CAP at dose ranges from 10 to 1000 μM for 48 h. **c**–**g** Cell apoptosis of CRC cells detected by flow cytometric analysis after treatment with 500 μM of CAP for 24 or 48 h. Data were expressed as the mean ± SD (n = 3) of three parallel experiments. ^*^P < 0.05, ^**^P < 0.01 vs control

Capecitabine (CAP) has been approved by the Food and Drug Administration (FDA) as a widely used oral chemotherapy agent for treating several malignancies, including CRC, gastric cancer, breast cancer and pancreatic cancer, as well as tumors known to be resistant to5-fluorouracil (5-FU) [7]. CAP is designed and developed as a tumor-selective prodrug, which is preferentially converted to the most active compound 5-FU in targeting tumor tissues via an enzymatic cascade involving three metabolic steps to elevate intra-tumoral drug concentrations. After oral administration, CAP is extensively absorbed through the intestine and metabolized to 5′-deoxy-5-fluorocytidine (5′-DFCR) by carboxylesterase (CES), an enzyme mainly located in the liver. 5'-DFCR is then converted to 5′-deoxy-5-fluorouridine (5′-DFUR) by cytidine deaminase (CyD), a ubiquitous enzyme with high activity in the liver and tumor tissues. Finally, 5'-DFUR is converted to the active and toxic metabolite 5-FU by thymidine phosphorylase (TP), which is 3–10 times more concentrated in various solid tumors than in normal adjacent tissues [8, 9] (Fig. 1a). The structural formulas of CAP and its metabolites were shown in Additional file 1: Fig. S1. The anti-tumor activity of 5-FU is generally attributed to the inhibition of de novo thymidine synthesis and incorporation into intracellular RNA or DNA [10].

Accordingly, previous clinical pharmacokinetic (PK) and pharmacodynamic (PD) studies of CAP demonstrated that intra-tumoral concentrations of 5-FU in patients with CRC following oral administration of CAP were at least 20 times higher than plasma and muscle concentrations, leading to subsequently equal clinical efficacy and reduced toxicity compared to 5-FU infusion regimens [11–13]. It also meant that plasma 5-FU concentrations following CAP administration did not reflect the actual concentrations in the targeted tissue. Additionally, it has been demonstrated that there is no clinically relevant correlation between several body size measures, such as body surface area (BSA) or body weight (which commonly guides dosage) and the PK profiles of CAP and its metabolites [14, 15]. Thus, it is necessary to develop a suitable and reliable model to explore the PK and PD properties of CAP and its metabolites, as the classical PK theory is based on the premise that plasma drug concentration is proportional to drug efficacy.

Monolayer culture is the most common and conventional in vitro cell model, with simplicity, stability, and low-cost advantages. However, a growing number of studies have suggested that cell metabolism, differentiation, function, even gene-expression profiles and metabolic profiles are significantly altered in monolayer culture [16–18]. Owing to the reduced amount of cell–cell communication and a less physiological cell shape, monolayer culture is much less similar to the in vivo environment. Therefore, in monolayer cell culture, it is not only challenging to simulate the real metabolic process of drugs in vivo, but also insufficient to study drug efficacy. A co-culture system is a cell cultivation device that allows contact between two or more distinct cell populations. It is frequently applied to better simulate the cell–cell interaction, even the natural physiological environment in vivo [19]. Co-cultures are considered more representative of human in vivo-like tissue models than animal models, and provide the possibility of high-throughput testing and in-depth monitoring for drug research [20, 21]. Guzzardi et al. [22] demonstrated altered cellular metabolic processes in HepG2 cells co-cultured with human umbilical vein EC cells (HUVECs). Choe et al. [23] successfully established a novel microfluidic enterohepatic microarray consisting of two separate layers for Caco-2 (gut epithelial cells) and HepG2 cells to reproduce the first-pass metabolism of flavonoids. Compared to gut monoculture cells, the metabolic profile of co-culture gut and liver cells was closer to the reported profile. Therefore, an in vitro co-culture model can provide insights into complex physiological and pathological conditions that cannot be recapitulated in a conventional monolayer culture.

As CAP requires activation through metabolic enzymes in the liver, a co-culture system of hepatocytes and CRC cells, instead of a conventional monolayer culture, was constructed to mimic the metabolic processes of CAP and employed in cellular PK and PD studies, as well as PK-PD correlation analysis. This was the first study to explore the cellular PK and PD characteristics of CAP and its metabolites during CAP treatment in vitro. Furthermore, we provided a reliable model for high-throughput efficacy evaluation and mechanistic research for prodrugs in vitro to better understand the underlying mechanisms of efficacy and toxicity.

## Materials and methods

### Reagents

CAP (purity, 99.73%) and tolbutamide (internal standard, IS) (purity, 99.88%) were purchased from MedChem Express (MCE, Shanghai, China). 5'-DFCR (purity ≥ 99%), 5'-DFUR (purity ≥ 98%) and 5-FU (purity ≥ 99%) were obtained from Shanghai yuanye Bio-Technology Co., Ltd (Shanghai, China), Abcam (Cambridge, UK) and Sigma-Aldrich (St. Louis, MO, USA), respectively. DMEM/F-12, RPMI 1640, L-15 Medium, fetal bovine serum (FBS), penicillin/streptomycin and trypsin were gained from GIBCO (Grand Island, NY, USA). Cell Counting Kit-8 (CCK-8), bicinchoninic acid (BCA) protein assay kit and protein isolation kit were obtained from Beyotime Biotechnology (Jiangsu, China). Annexin V-fluorescein isothiocyanate (FITC)/propidium iodide (PI) staining Apoptosis Detection kit and enhanced chemiluminescence (ECL) kit were purchased from KeyGen Biotech (Nanjing, Jiangsu, China).

### Cell culture, treatment, and model construction

Five human CRC cell lines, LoVo (DMEM/F-12), HCT-8 (RPMI-1640), HCT-116 (DMEM), SW480 (L-15), SW620 (RPMI-1640) and human hepatocarcinoma cell line, HepG2 (DMEM) were obtained from National Collection of Authenticated Cell Cultures (Shanghai, China). All cell lines were cultured in culture media supplemented with 10% (v/v) FBS and 1% (v/v) penicillin/streptomycin, and incubated in 5% CO_2_ at 37 °C in a water saturated atmosphere and sub-cultured every three days. STR profiling and mycoplasma contamination were performed to keep the authenticity of cell line on regular basis. Then, CRC cells were used to construct Model 1 (pre-metabolized by HepG2 cells) and Model 2 (co-cultured with HepG2 cells).

#### Model 1 (pre-metabolized by HepG2 cells)

For Model 1 construction, HepG2 cells were seeded in 6-well plates, and incubated with the medium containing CAP for 0, 24 and 48 h after adhesion. The medium metabolized by HepG2 cells in each group was collected and added to 24-well plates with CRC cells for the following treatments.

#### Model 2 (co-cultured with HepG2 cells)

For Model 2 construction, HepG2 cells were seeded in 0.4 µm transwell inserts (upper compartment) and CRC cells were seeded in 6-well plates. After adhesion, a co-culture system was established in which each transwell insert (an upper compartment with HepG2 cells) was inserted into each well (a lower compartment with CRC cells) of 6-well plates. Then, co-cultures of HepG2 and CRC cells were incubated with the medium containing CAP.

### Cell viability assay

According to the manufacturer’s instructions, cell viability was estimated using CCK-8 kit. Briefly, cells were seeded into cell culture cates at a suitable density per well. After adhesion, the experimental groups were treated with 5-FU or CAP at the indicated administration. After treatment, cells were washed with PBS twice and incubated with 100 μL non-phenol red medium containing 10% CCK-8 solution in each well at 37 °C for an additional 2 h. An automatic microplate reader measured the absorbance at an OD of 450 nm.

### Flow cytometric analysis of cell apoptosis

After treated with 5-FU or CAP at the indicated administration, approximately 1 × 10^6^ cells per group were collected and centrifuged at 3, 000 rpm for 5 min and washed with PBS. Subsequently, cells were stained using an Annexin V FITC/PI staining Apoptosis Detection kit according to the manufacturer’s instructions. Briefly, annexin V-FITC and PI were added to cell suspension in buffer Binding. The cells were incubated at room temperature and kept away from light for 20–30 min, and then the cells were resuspended. The percentage of apoptotic cells for each sample was analyzed by Novocyte Advanteon flow cytometer (Agilent, USA) and NovoExpress software 14.1.

### Western blotting

Cells were resuspended in lysis buffer (RIPA) and protease inhibitor (PMSF) at 100:1. Protein contents in lysates were determined using BCA Assay Kit (Bio-Rad, Hercules, CA, USA) before adding sample buffer. Equal amounts of protein (50 µg/lane) were subjected to 12% sodium dodecyl sulfate–polyacrylamide gel (SDS-PAGE, Bio-Rad), and transferred to polyvinylidene fluoride (PVDF, Bio-Rad, 2.2 μm) membranes. The blotted membranes were blocked with 5% nonfat milk for 1 h at room temperature and incubated overnight with primary antibodies at 4 °C. Then they were incubated with HRP-conjugated secondary antibodies for 1 h at 37 °C. GAPDH was immunoblotted as an internal control. Finally, the protein bands were visualized using ECL kit and quantified using ImageJ v.1.8.0 software (National Institutes of Health). The following were commercially obtained antibodies: anti-GAPDH, anti-Bcl-2, anti-Bax, anti-cleaved caspase-3, anti-cleaved caspase-7, and anti-PARP1 antibodies were purchased from Cell Signaling Technology (CST, MA, USA). The dilution of all the primary antibody incubation in immunoblotting is 1:1000. Horseradish peroxidase (HRP) conjugated secondary antibodies mouse or rabbit IgG were purchased from Proteintech (Wuhan, Hubei, China).

### Cellular pharmacokinetics

#### Sample preparation

According to *2.2.1* (Model 1), HepG2 cells were incubated with the medium containing 500 μM of CAP for 0, 24 and 48 h after adhesion. And then the medium containing CAP and its metabolites metabolized by HepG2 in each group was added to 24-well plates with CRC cells for following 0, 1, 2, 4, 6, 12, 24 and 48 h. According to *2.2.2* (Model 2), co-culture systems of HepG2 and CRC cells were treated with 500 μM of CAP for 48 h. At the end of the experiment, treated CRC cells (in models of pre-metabolism by HepG2 or co-culture with HepG2) were washed with PBS and lysed in 300 μL of ddH_2_O. After repeatedly frozen and thawed three times between room temperature and − 80 °C [24], the cell suspension was collected and 100 µL was used for the protein concentration measurement with a BCA protein assay kit. 100 μL of cell lysate was transferred into a 1.5 mL Eppendorf tube. 400 μL of the de-protein solvent containing 200 ng/mL of tolbutamide (IS) was added to each cell lysate sample and then vortexed for 5 min. The mixture samples were centrifuged (14,000 rpm × 5 min, 4 °C) and 200 μL of supernatant of each sample was transferred to another Eppendorf tube. After centrifugation (14,000 rpm × 10 min, 4 °C) again, 100 μL of the supernatant was collected and injected into an auto-sampler bottle.

#### Liquid chromatography–tandem mass spectrometry technique (LC–MS/MS) method establishment and validation

The LC–MS/MS system consisted of a high-performance liquid chromatography (HPLC) system (Shimadzu DGU-20AD XR system Tokyo, Japan) and a triple quadruple mass spectrometer (AB SCIEX 5500-QTrap®, CA, USA). Data acquisition and processing were performed with Analyst 1.5.2 software (AB SCIEX, CA, USA). Chromatographic separation was achieved on an HP Amide C18-ODS column (10 cm × 3 mm, 5 μm). The mobile phase consisted of H_2_O (solvent A), and acetonitrile (solvent B) at a flow rate of 0.2 mL/min. The elution gradient was as 50.0% solvent B maintained for 3 min, and the injection volume was set at 10 μL. The column temperature was 40 °C. Before used, mobile phase A was filtered through 0.45 pore size cellulose nitrate membrane filters using a vacuum filtration apparatus. The samples were kept in the autosampler at 4 °C throughout the analysis process. The needle rinse solution was acetonitrile.

The mass spectrometer was operated with a TurboIonSpray™ source in the negative ion mode. Multiple-reaction monitoring (MRM) was used to monitor analyte and tolbutamide (IS) with the source temperature of 550 °C, Ion Source Gas1 was 55 psi, Ion Source Gas2 was 35 psi, IonSpray Voltage was 4500 V, Curtain Gas was 40 psi, collision gas, medium. The selected mass transitions were m/z 358.1 → 153.9 for CAP, m/z 243.9 → 126.9 for 5′-DFCR, m/z 244.9 → 107.9 for 5′-DFUR, m/z 128.9 → 42.0 for 5-FU, m/z 500.9 → 158.9 for 5-fluorouridine 5′-triphosphate (FUTP), m/z 484.8 → 256.8 for 5-fluoro-2′-deoxyuridine 5′-triphosphate (FdUTP), m/z 325.0 → 128.9 for 5-fluoro-2′-deoxyuridine 5′-monophosphate (FdUMP) and m/z 268.9 → 169.9 for tolbutamide (IS) (Additional file 2: Fig. S6). The “LC–MS/MS method validation” section, including specificity, linearity, accuracy, precision, matrix effect, recovery, stability and dilution integrity was supplied in Additional file 2: Fig. S6–S8 and Tables S2–S6.

### Statistical analysis

The pharmacokinetic parameters based on a non-compartmental statistical model were calculated using the Phoenix WinNonlin pharmacokinetic program (Version 7.0, Pharsight, Mountain View, CA). Experiments were repeated at least three times independently and data were expressed as mean ± standard deviation (SD) for each group. Statistical analyses were conducted by two-tailed Student’s t-test and one-way ANOVA. The difference was considered statistically significant when the probability value was less than 0.05 (*P* < 0.05).

## Results

### Conventional monolayer cultures are insufficient for PK and PD studies of CAP

To determine whether different CRC cell lines showed distinct sensitivities, the efficacy of CAP and its active metabolite, 5-FU at various doses were examined on five monolayer CRC cell lines (LoVo, SW480, HCT-8, HCT116, and SW620). CAP exhibited little anti-proliferative effect on all monolayer CRC cells in the dosage range of 10–1000 μM (Fig. 1b, Additional file 1: Fig. S2). Similarly, 500 μM of CAP did not induce apoptosis in monolayer CRC cells after 24 h of treatment (*P* > 0.05). However, when the treatment time was extended to 48 h, the apoptosis rates of the five CRC cell lines were increased a little in the CAP group (*P* < 0.05, *P* < 0.01; Fig. 1c–g).

**Fig. 2:**
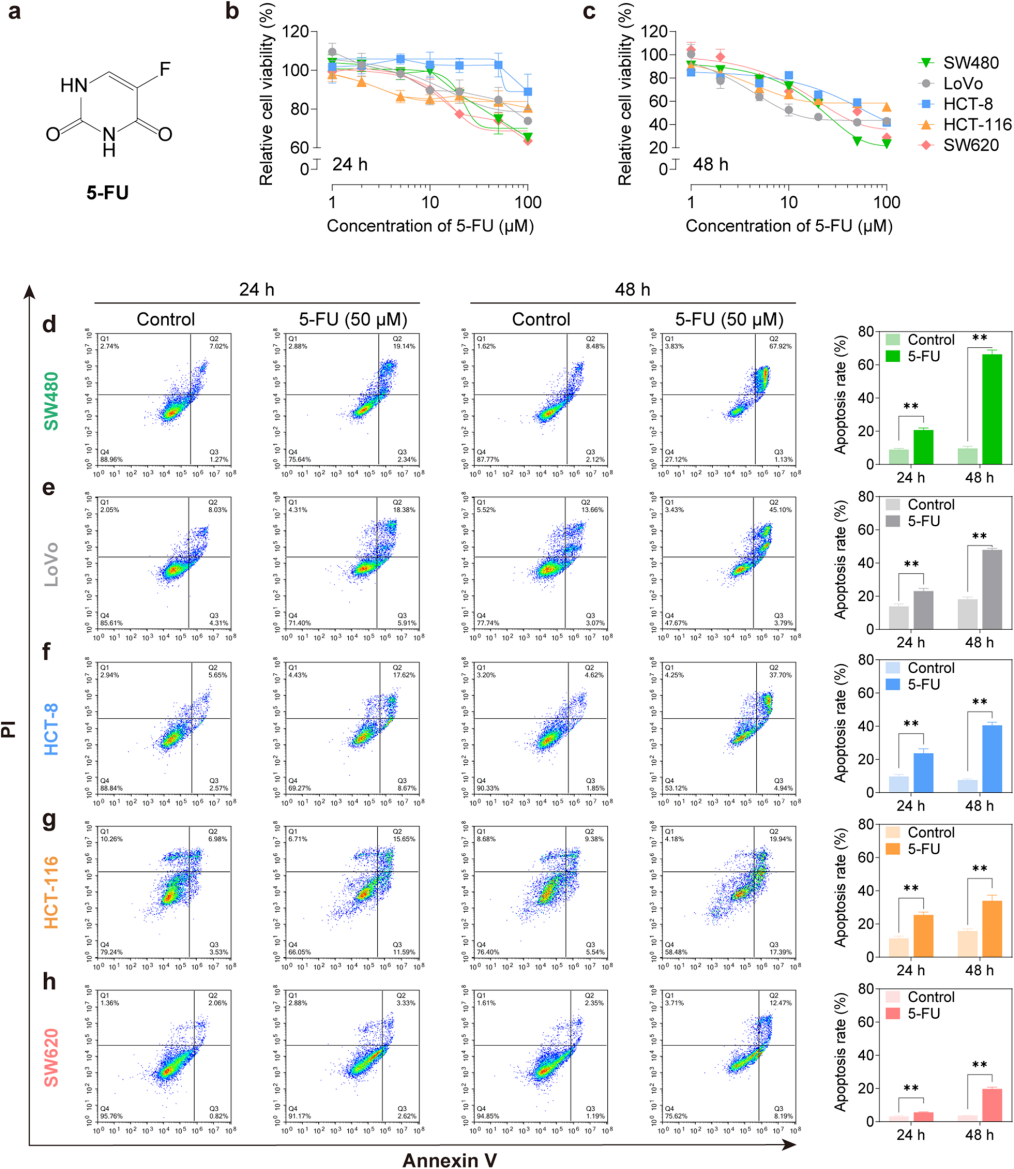
5-FU significantly inhibited cell viability and induced cell apoptosis in CRC cells. **a**–**b** Relative cell viability of CRC cells measured by the CCK-8 assay after treatment with 5-FU at various doses ranging from 1 to 100 μM for 24 or 48 h. **c**–**g** Cell apoptosis of CRC cells detected by flow cytometric analysis after treatment with 50 μM of 5-FU for 24 or 48 h. Data were expressed as the mean ± SD (n = 3) of three parallel experiments. ^*^P < 0.05, ^**^P < 0.01 vs control

Subsequently, we evaluated the inhibitory effects of 5-FU (Fig. 2a), an active metabolite of CAP on cell growth. Unlike CAP, 5-FU showed obvious cytotoxicity on monolayer CRC cells in a dose-dependent manner (Fig. 2b, c, Additional file 1: Fig. S2). We treated monolayer CRC cells with 50 μM of 5-FU for 24 and 48 h, and found that 5-FU significantly promoted apoptosis in all monolayer CRC cells (*P* < 0.01; Fig. 2d–g). Furthermore, 5-FU displayed varied efficacy across different CRC cell lines, which could be classified as follows: SW480 and LoVo cells were highly susceptible to 5-FU, and defined as high-sensitive type (Fig. 2d); HCT-8 and HCT-116 were intermediately susceptible to 5-FU, and defined as medium-sensitive type (Fig. 2e, f); whereas SW620 was nearly insensitive to 5-FU, and appeared to be drug-resistant type (Fig. 2g). These results revealed that CAP must be activated through hepatic metabolism to exert its anti-tumor activity. It was necessary to build a suitable cell model, rather than a simple monolayer cell model to explore the pharmaceutical properties of CAP.

### Establishment of a pre-metabolism model by hepatocytes for the pharmacodynamic study of CAP

Primary hepatic parenchymal cells, HepG2 cells, and human-derived normal hepatocytes (HepaRG cells) have the closest metabolic capacity to that of primary hepatocytes which are commonly used hepatocyte models. Due to the difficulty in obtaining HepaRG cells, we examined the basal metabolic and efflux capacity of primary mouse hepatocytes and HepG2 cells on CAP to select an appropriate hepatocyte model for in vitro research. After treatment with 500 μM of CAP, primary mouse hepatocytes and HepG2 cells were collected at different time points and analyzed by LC–MS/MS for the concentrations of 5'-DFCR, 5'-DFUR, and 5-FU in the cell culture medium. Concentration–time profiles in the culture medium of primary mouse hepatocytes and HepG2 cells were shown in Additional file 1: Fig. S3. The main PK parameters of the maximum concentration (C_max_), time to reach maximum concentration (T_max_), and area under the concentration time curve from zero to the time of last measurable concentration (AUC_0-t_) of the five CRC cell lines were presented in Additional file 1: Table S1. Values of C_max_ and AUC_0-t_ of 5-FU in the culture medium of HepG2 cells were 0.17 ± 0.007 μg/mL and 3.01 ± 0.12 h*μg/mL, respectively, and the values of C_max_ and AUC_0-t_ of 5-FU in the culture medium of primary mouse hepatocytes were 0.10 ± 0.005 μg/mL and 2.25 ± 0.21 h*μg/mL, respectively. The capacity of HepG2 cells to metabolize CAP and excrete 5-FU was significantly enhanced compared to primary mouse hepatocytes (*P* < 0.05; Additional file 1: Table S1). Considering the poor efficiency, significant variation between lots, and the high difficulty of acquiring primary hepatocytes, HepG2 cells were used in our study.

**Fig. 3:**
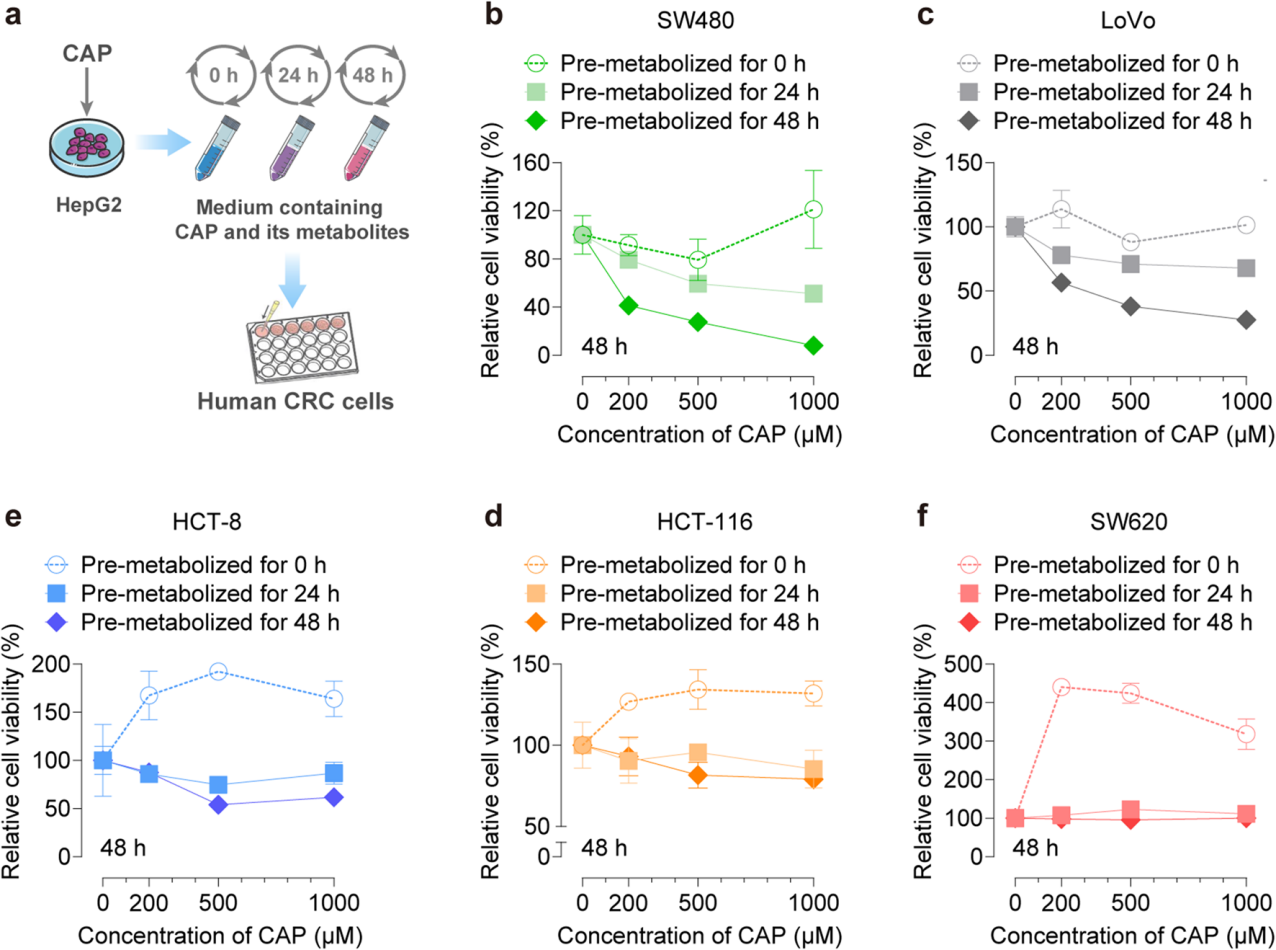
CAP showed the cytotoxic effect on the growth of CRC cells after pre-metabolized by HepG2 cells. **a** Schematic workflow to establish the model of pre-metabolism by HepG2 cells. First, HepG2 cells were treated with the indicated doses of CAP for 0, 24 or 48 h. Then the medium metabolized by HepG2 cells in each group was collected and added to CRC cells for the following treatments. **b**–**f** After HepG2 cells were treated with 200, 500 or 1000 μM of CAP for 0, 24 or 48 h, the medium of each group was collected and added to CRC cells for the following 48 h treatment. Relative cell viability of CRC cells was measured by the CCK-8 assay. Data were expressed as the mean ± SD (n = 3) of three parallel experiments

To demonstrate the critical role of the liver in the metabolic activation of CAP, we evaluated the anticancer activity of CAP at various doses in five CRC cell lines after pre-metabolized by HepG2 cells for 0, 24 and 48 h. As shown in Fig. 3a, HepG2 cells were incubated with the medium containing 0, 200, 500, or 1000 μM of CAP for 0, 24, or 48 h respectively. Then the medium containing CAP and its metabolites in each group was collected and added to CRC cells for the following 48 h of treatment. Without pre-metabolized by HepG2 cells (pre-metabolized for 0 h), CAP showed a slight anti-tumor effect on SW480 and LoVo cells at an initial concentration of 200 or 500 μM. However, CAP exhibited a pro-proliferative effect at a concentration of 1000 μM. Under pre-metabolized by HepG2 cells for 24 or 48 h, CAP inhibited the growth of SW480 and LoVo cells in a dose-dependent manner (Fig. 3b, c). Similarly, CAP promoted the proliferation of HCT-8 and HCT-116 cells under pre-metabolized by HepG2 cells for 0 h. After pre-metabolized by HepG2 cells for 24 or 48 h, a slight inhibitory effect of CAP was observed (Fig. 3d, e). For SW620 cells, CAP demonstrated a strong proliferative activity, although this effect was diminished with little cytotoxicity under pre-metabolized by HepG2 cells (Fig. 3f). These findings suggested that the anti-tumor effects of CAP were significantly enhanced after pre-metabolized by HepG2 cells, and that CAP showed differential efficacy across five CRC cell lines when compared with monolayer CRC cell lines, indicating differential metabolic conversion to active metabolites.

### Establishment of a co-culture system of liver and intestinal cells for the PD study of CAP

**Fig. 4:**
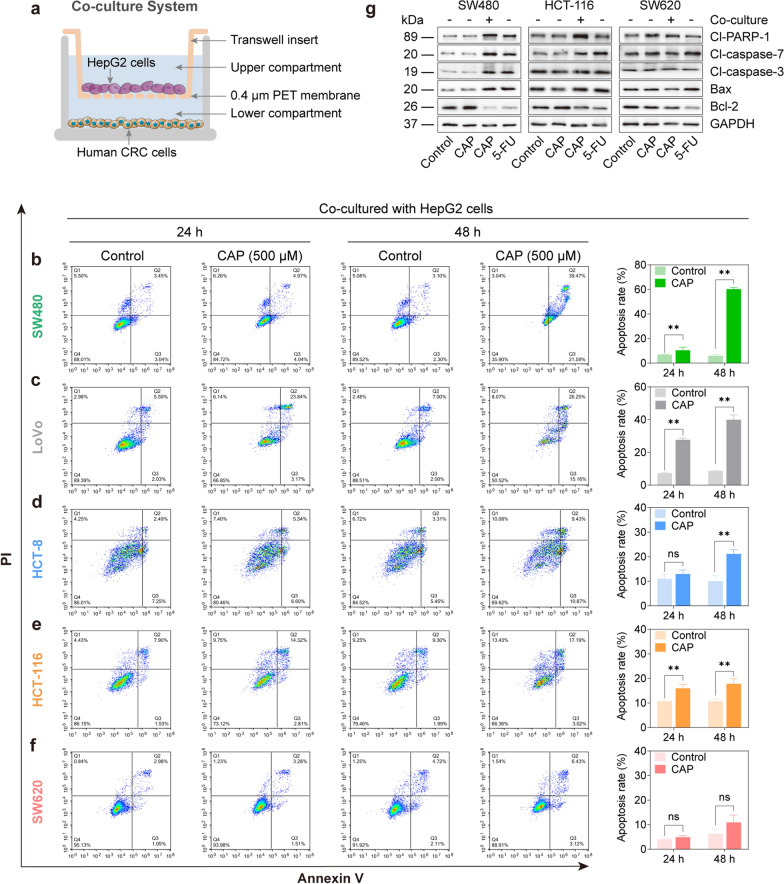
CAP showed the cytotoxic effect on the growth of CRC cells under co-cultured with HepG2 cells. **a** Schematic workflow to establish the co-culture system of CRC and HepG2 cells with HepG2 cells in the upper compartment and CRC cells in the lower compartment. Then, the established co-culture system was incubated with the medium containing CAP. **b**–**f** Cell apoptosis of CRC cells under co-cultured with HepG2 cells detected by flow cytometric analysis after treatment with 500 μM of CAP for 24 or 48 h. **g** Apoptosis-related protein levels detected by western blotting in CRC cells after treatment with 500 μM of CAP under co-culture with HepG2 cells or not, and 50 μM of 5-FU for 48 h. Data were expressed as the mean ± SD (n = 3) of three parallel experiments. ^*^P < 0.05, ^**^P < 0.01 vs control. Abbreviations: DHFU, dihydrofluorouracil; FUPA, α-fluoro-β-ureidopropionic acid; FBAL, α-fluoro-β-alanine; FUMP, 5-fluorouridine 5′-monophosphate; FUDP, 5-fluorouridine 5′-diphosphate; FUTP, 5-fluorouridine 5′-triphosphate; FdUMP, 5-fluoro-2′-deoxyuridine 5′-monophosphate; FdUDP, 5-fluoro-2′-deoxyuridine 5′-diphosphate; FdUTP, 5-fluoro-2′-deoxyuridine; dTMP, 2′-deoxythymidine 5′-monophosphate; dUMP, 2′-deoxyurdine 5′-monophosphate; dUDP, 2′-deoxyurdine 5′-diphosphate; dUTP, 2′-deoxyurdine 5′-triphosphate

To further elucidate the function of metabolic activation by hepatocytes in the anti-tumor effect of CAP on CRC cell lines, a co-culture system of HepG2 and CRC cells was established. As shown in Fig. 4a, HepG2 cells were cultured in transwell inserts in the upper compartment and CRC cells were cultured in the lower compartment, respectively. Then, co-cultures of HepG2 and CRC cells were incubated with the medium containing 500 μM of CAP for 24 or 48 h and apoptosis of CRC cells was measured. We found that CAP triggered apoptosis of CRC cells in co-cultures with HepG2 cells (Fig. 4b–f), compared to the weak efficacy of CAP in monolayer cells (Fig. 1b–g). Furthermore, different CRC cell lines in co-cultures displayed differential susceptibilities to CAP. Consistent with the differential efficacy of 5-FU in monolayer CRC cells (Fig. 2b–f), SW480 and LoVo cells were sensitive, HCT-8 and HCT-116 cells were intermediately sensitive, and SW620 cells were resistant to CAP treatment under co-cultured with HepG2 cells. Three cell lines with different sensitivities, including SW480 (sensitive), HCT-116 (intermediately sensitive) and SW620 (resistant) were selected to evaluate the expression of apoptosis-related proteins after treatment with CAP or 5-FU. We found that CAP had little effect on apoptosis-related protein expressions in three monolayer cell lines (Fig. 4g, Additional file 1: Fig. S4), consistent with the findings shown in Fig. 1c, e and g. In SW480 (sensitive) cells, 5-FU (in monolayer cells) and CAP (in a co-culture system) dramatically down-regulated Bcl-2 (an anti-apoptotic protein) and highly up-regulated pro-apoptotic proteins, including Bax, Caspase-7, Caspase-3 and PARP-1. In HCT-116 (intermediately sensitive) cells, anti- and pro-apoptotic proteins were moderately regulated after treatment with 5-FU (in monolayer cells) and CAP (in co-culture system). In contrast, the expressions of apoptosis-related proteins were slightly affected in SW620 (resistant) cells (Fig. 4g, Additional file 1: Fig. S4). These findings revealed that CRC and HepG2 cells could be successfully co-cultured and the efficacy of CAP were significantly improved in the established co-cultures compared to monolayer CRC cell lines, suggesting that CAP was successfully activated and metabolized to active metabolites.

### Cellular PK profiles of CAP in CRC cells under pre-metabolized by HepG2 cells

CAP is activated through the metabolism in the liver and tumor cells in vivo, and is then converted into active metabolites to exert its antitumor effect (Fig. 1a). Based on this, we wondered whether the differential activities of CAP against the five CRC cell lines were correlated with the differences in PK behaviors. We also investigated the involved mechanisms contributing to the differential efficacy of CAP from the perspective of cellular PK characteristics.

**Fig. 5:**
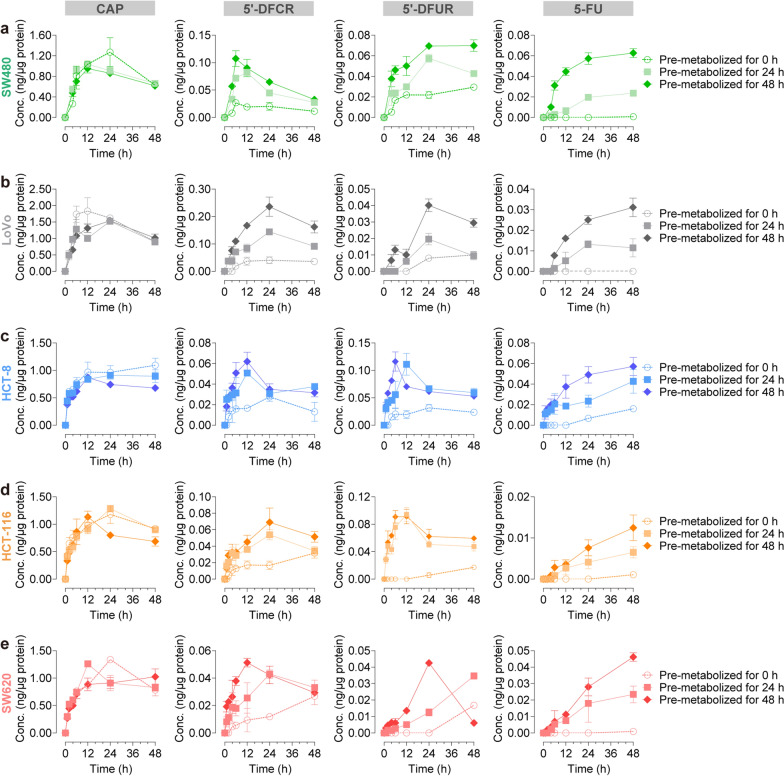
Cellular pharmacokinetic profiles of CAP, 5′-DFCR, 5′-DFUR and 5-FU in CRC cells after pre-metabolized by HepG2 cells. After HepG2 cells were treated with 500 μM of CAP for 0, 24 or 48 h, the medium of each group was collected and added to CRC cells. For following 0, 2, 4, 6, 12, 24, and 48 h treatments, intracellular concentration–time curves of CAP, 5′-DFCR, 5′-DFUR and 5-FU were plotted. Each point represents mean ± SD (n = 4)

Using tolbutamide as the internal standard (IS), we established a stable and reliable LC–MS/MS method for the four quantitative (5'-DFCR, 5'-DFUR and 5-FU) and three semi-quantitative (FUTP, FdUTP, and FdUMP) detection of CAP and its metabolites simultaneously (Additional file 2: Fig*.* S6–S8 and Tables S2–S6). We evaluated the cellular PK behaviors of CAP in five CRC cell lines under pre-metabolized by HepG2 cells. As shown in Fig. 3a, HepG2 cells were incubated with the medium containing 500 μM of CAP for 0, 24, and 48 h. Then, the medium containing CAP and its metabolites in each group was collected and added to CRC cells for following 0, 1, 2, 4, 6, 12, 24, and 48 h. Using LC–MS/MS method, concentration–time curves of CAP, 5′-DFCR, 5′-DFUR and 5-FU in the five CRC cell lines were shown in Fig. 5a–e. The PK parameters calculated by the Phoenix WinNonlin PK program were shown in Tables 1, 2, 3, 4 and 5, respectively. It was demonstrated that the intervention of hepatocytes could activate the metabolic process and significantly increase the concentration and exposure of 5-FU in CRC cells (Fig. 5a–e). The C_max_ and AUC_0-t_ of intracellular 5-FU after pre-metabolized for 24 h ranged from 1.4 to 25 times and 1.9 to 180 times respectively, that in the absence of HepG2 cells (pre-metabolized by HepG2 cells for 0 h). The C_max_ and AUC_0-t_ of intracellular 5-FU after pre-metabolized for 48 h ranged from 3 to 75 times and 4 to 260 times respectively, of those in the absence of HepG2 cells (pre-metabolized by HepG2 cells for 0 h), in which cellular PK behaviors were most promoted in SW480 cells (Tables 1, 2, 3, 4 and 5). Hence, the peak concentration and exposure of 5-FU increased significantly after 48 h of pre-metabolism compared to 24 h of pre-metabolism by HepG2 cells (Fig. 5a–e). These results indicated that the cellular PK profiles of CAP and its metabolites in five CRC cell lines were remarkably improved under pre-metabolized by HepG2 cells, suggesting that hepatocellular intervention was of great significance for the metabolic activation of CAP.

**Table 1:** Pharmacokinetic parameters of CAP and its metabolites (5'-DFCR, 5'-DFUR and 5-FU) in SW480 cells after CAP administration (500 μM) (Mean ± SD, n = 4)

SW480		T_max_ (h)	C_max_ (ng/μg protein)	AUC_0-t_ (h*ng/μg protein)
Pre-metabolized by HepG2 cells for 0 h	CAP	16.00 ± 6.93	1.27 ± 0.27	43.77 ± 2.18
	5'-DFCR	6.00 ± 0	0.03 ± 0.002	0.81 ± 0.07
	5'-DFUR	48 ± 0	0.03 ± 0.001	1.03 ± 0.09
	5-FU	48 ± 0	0.0008 ± 0.00005	0.009 ± 0.0003
Pre-metabolized by HepG2 cells for 24 h	CAP	12 ± 0^**^	1.04 ± 0.08	39.05 ± 3.36
	5'-DFCR	12 ± 0^**^	0.08 ± 0.003*^*^	2.27 ± 0.17^**^
	5'-DFUR	24 ± 0^**^	0.06 ± 0.005^**^	1.98 ± 0.06^**^
	5-FU	48 ± 0	0.02 ± 0.002^**^	0.72 ± 0.05^**^
Pre-metabolized by HepG2 cells for 48 h	CAP	12 ± 0^**^	0.95 ± 0.08^**^	35.74 ± 5.89^**^
	5'-DFCR	6 ± 0	0.11 ± 0.05^**^	2.97 ± 0.66^**^
	5'-DFUR	48 ± 0	0.07 ± 0.004^**^	2.84 ± 0.51^**^
	5-FU	48 ± 0	0.06 ± 0.007^**^	2.34 ± 0.44^**^

*T*_*max*_ time to peak concentration; *C*_*max*_ peak concentration; *AUC*_*0-t*_ area under the concentration–time curve from zero to the time of last measurable concentration

^*^P < 0.05

^**^P < 0.01 compared with the group of pre-metabolized by HepG2 cells for 0 h

**Table 2:** Pharmacokinetic parameters of CAP and its metabolites (5'-DFCR, 5'-DFUR and 5-FU) in LoVo cells after CAP administration (500 μM) (Mean ± SD, n = 4)

LoVo		T_max_ (h)	C_max_ (ng/μg protein)	AUC_0-t_ (h*ng/μg protein)
Pre-metabolized by HepG2 cells for 0 h	CAP	12 ± 0	1.83 ± 0.39	66.33 ± 5.55
	5'-DFCR	24 ± 0	0.41 ± 0.11	1.57 ± 0.42
	5'-DFUR	48 ± 0	0.01 ± 0.003	0.27 ± 0.01
	5-FU	48 ± 0	0.007 ± 0.0002	0.23 ± 0.03
Pre-metabolized by HepG2 cells for 24 h	CAP	24 ± 0^**^	1.52 ± 0.76^*^	55.24 ± 6.64^**^
	5'-DFCR	24 ± 0	0.14 ± 0.04^**^	4.89 ± 0.88^**^
	5'-DFUR	24 ± 0^**^	0.02 ± 0.001^**^	0.52 ± 0.09^**^
	5-FU	24 ± 0^**^	0.01 ± 0.003^*^	0.43 ± 0.02^**^
Pre-metabolized by HepG2 cells for 48 h	CAP	24 ± 0^**^	1.54 ± 0.35^*^	57.96 ± 0.74^*^
	5'-DFCR	24 ± 0	0.24 ± 0.03^**^	8.37 ± 0.53^**^
	5'-DFUR	24 ± 0^**^	0.04 ± 0.002^**^	1.24 ± 0.24^**^
	5-FU	48 ± 0	0.03 ± 0.009^**^	1.00 ± 0.09^**^

*T*_*max*_ time to peak concentration; *C*_*max*_ peak concentration; *AUC*_*0-t*_ area under the concentration–time curve from zero to the time of last measurable concentration

^*^P < 0.05

^**^P < 0.01 compared with the group of pre-metabolized by HepG2 cells for 0 h

**Table 3:** Pharmacokinetic parameters of CAP and its metabolites (5'-DFCR, 5'-DFUR and 5-FU) in HCT-8 cells after CAP administration (500 μM) (Mean ± SD, n = 4)

HCT-8		T_max_ (h)	C_max_ (ng/μg protein)	AUC_0-t_ (h*ng/μg protein)
Pre-metabolized by HepG2 cells for 0 h	CAP	48 ± 0	1.09 ± 0.18	44.88 ± 5.61
	5'-DFCR	24 ± 0	0.03 ± 0.01	0.91 ± 0.05
	5'-DFUR	24 ± 0	0.03 ± 0.009	1.14 ± 0.73
	5-FU	48 ± 0	0.02 ± 0.002	0.31 ± 0.01
Pre-metabolized by HepG2 cells for 24 h	CAP	24 ± 0^**^	0.91 ± 0.05	40.10 ± 3.41
	5'-DFCR	12 ± 0^**^	0.05 ± 0.006^**^	1.71 ± 0.78^**^
	5'-DFUR	12 ± 0^**^	0.11 ± 0.07^**^	3.32 ± 0.44^**^
	5-FU	48 ± 0	0.043 ± 0.003^**^	1.25 ± 0.77^**^
Pre-metabolized by HepG2 cells for 48 h	CAP	12 ± 0^**^	0.87 ± 0.09^*^	33.94 ± 6.12^**^
	5'-DFCR	12 ± 0^**^	0.062 ± 0.002^**^	1.90 ± 0.34^**^
	5'-DFUR	6 ± 0^**^	0.12 ± 0.07^**^	3.12 ± 0.17^**^
	5-FU	48 ± 0	0.06 ± 0.005^**^	2.07 ± 0.22^**^

*T*_*max*_ time to peak concentration; *C*_*max*_ peak concentration; *AUC*_*0-t*_ area under the concentration–time curve from zero to the time of last measurable concentration

^*^P < 0.05

^**^P < 0.01 compared with the group of pre-metabolized by HepG2 cells for 0 h

**Table 4:** Pharmacokinetic parameters of CAP and its metabolites (5'-DFCR, 5'-DFUR and 5-FU) in HCT-116 cells after CAP administration (500 μM) (Mean ± SD, n = 4)

HCT-116		T_max_ (h)	C_max_ (ng/μg protein)	AUC_0-t_ (h*ng/μg protein)
Pre-metabolized by HepG2 cells for 0 h	CAP	24 ± 0	1.18 ± 0.55	47.15 ± 3.31
	5'-DFCR	48 ± 0	0.032 ± 0.007	0.92 ± 0.04
	5'-DFUR	48 ± 0	0.02 ± 0.001	0.31 ± 0.08
	5-FU	48 ± 0	0.001 ± 0.0005	0.01 ± 0.001
Pre-metabolized by HepG2 cells for 24 h	CAP	24 ± 0	1.28 ± 0.4	47.67 ± 2.21
	5'-DFCR	24 ± 0^**^	0.05 ± 0.007^**^	1.93 ± 0.33^**^
	5'-DFUR	12 ± 0^**^	0.09 ± 0.005^**^	2.82 ± 0.17^**^
	5-FU	48 ± 0	0.007 ± 0.0003^**^	0.18 ± 0.03^**^
Pre-metabolized by HepG2 cells for 48 h	CAP	12 ± 0^**^	1.13 ± 0.02	38.63 ± 2.20^**^
	5'-DFCR	24 ± 0^**^	0.07 ± 0.004^**^	2.52 ± 0.11^**^
	5'-DFUR	12 ± 0^**^	0.09 ± 0.003^**^	3.26 ± 0.14^**^
	5-FU	48 ± 0	0.01 ± 0.0005^**^	0.33 ± 0.06^**^

*T*_*max*_ time to peak concentration; *C*_*max*_ peak concentration; *AUC*_*0-t*_ area under the concentration–time curve from zero to the time of last measurable concentration

^*^P < 0.05

^**^P < 0.01 compared with the group of pre-metabolized by HepG2 cells for 0 h

**Table 5:** Pharmacokinetic parameters of CAP and its metabolites (5'-DFCR, 5'-DFUR and 5-FU) in SW620 cells after CAP administration (500 μM) (Mean ± SD, n = 4)

SW620		T_max_ (h)	C_max_ (ng/μg protein)	AUC_0-t_ (h*ng/μg protein)
Pre-metabolized by HepG2 cells for 0 h	CAP	24 ± 0	1.34 ± 0.23	46.54 ± 2.27
	5'-DFCR	48 ± 0	0.03 ± 0.004	0.65 ± 0.03
	5'-DFUR	48 ± 0	0.02 ± 0.003	0.20 ± 0.05
	5-FU	48 ± 0	0.001 ± 0.0004	0.01 ± 0.004
Pre-metabolized by HepG2 cells for 24 h	CAP	12 ± 0^**^	0.001 ± 0.0004	42.93 ± 1.54
	5'-DFCR	24 ± 0^**^	0.04 ± 0.001	1.54 ± 0.32^**^
	5'-DFUR	48 ± 0	0.03 ± 0.002^*^	0.70 ± 0.01^**^
	5-FU	48 ± 0	0.007 ± 0.0003^**^	0.70 ± 0.02^**^
Pre-metabolized by HepG2 cells for 48 h	CAP	48 ± 0^**^	1.02 ± 0.23^*^	41.45 ± 1.36^*^
	5'-DFCR	12 ± 0^**^	0.05 ± 0.003^**^	1.83 ± 0.44^**^
	5'-DFUR	24 ± 0^**^	0.04 ± 0.007^**^	1.01 ± 0.26^**^
	5-FU	48 ± 0	0.05 ± 0.004^**^	1.20 ± 0.37^**^

*T*_*max*_ time to peak concentration; *C*_*max*_ peak concentration; *AUC*_*0-t*_ area under the concentration–time curve from zero to the time of last measurable concentration

^*^P < 0.05

^**^P < 0.01 compared with the group of pre-metabolized by HepG2 cells for 0 h

### *Cellular PK profiles of CAP in CRC cells under co-cultured with HepG2 cell*s

**Fig. 6:**
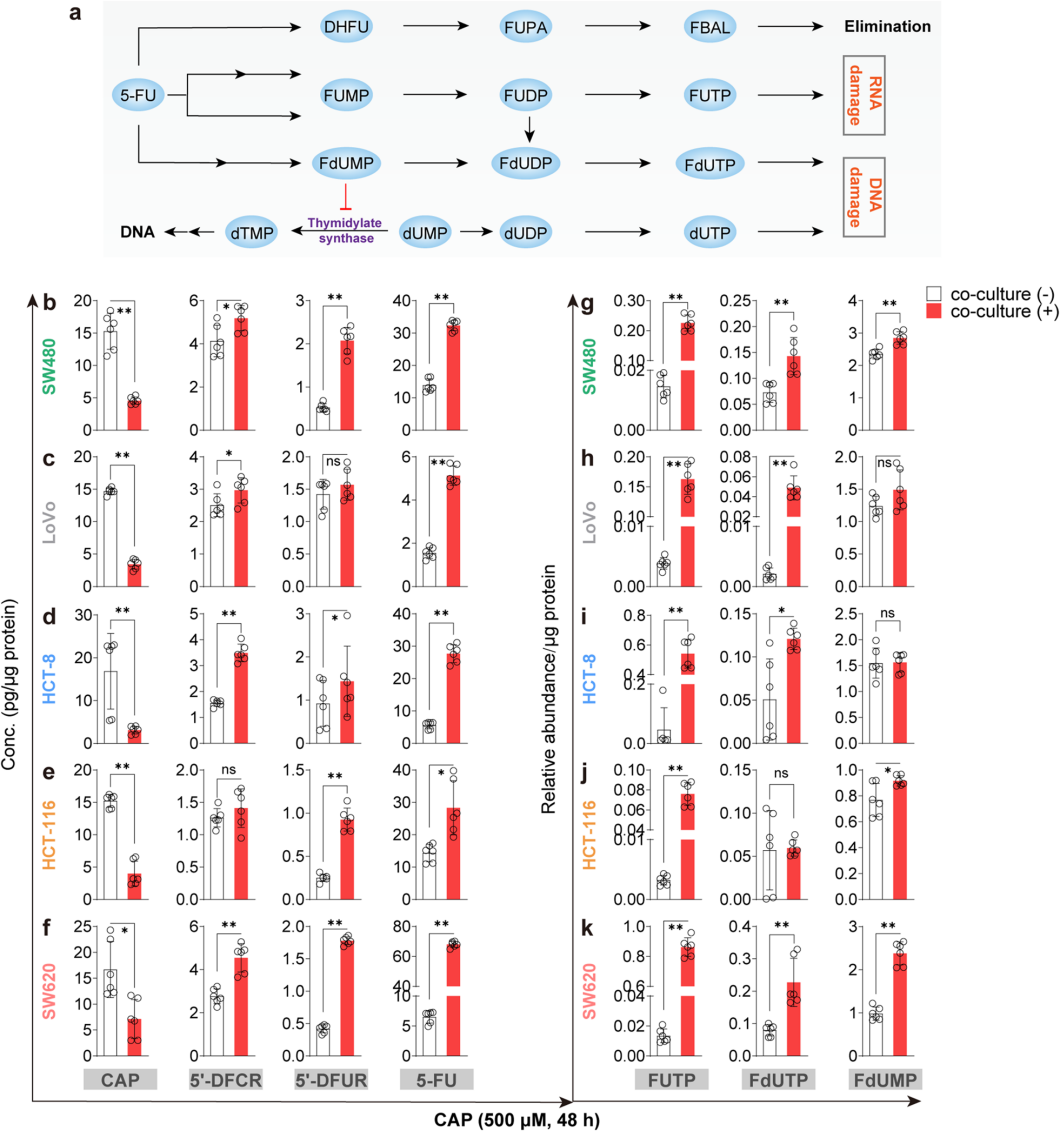
Intracellular concentrations of CAP, 5′-DFCR, 5′-DFUR, 5-FU, FUTP, FdUTP and FdUMP in CRC cells under co-cultured with HepG2 cells or not. **a** Metabolic pathway of 5-FU. **b**–**f** Intracellular concentrations of CAP, 5′-DFCR, 5′-DFUR, 5-FU, FUTP, FdUTP and FdUMP under co-culture or not. Each point represents mean ± SD (n = 6). ^*^P < 0.05, ^**^P < 0.01 vs the group without co-culture

We further investigated the cellular PK characteristics of CAP in co-culture systems of five CRC cell lines with HepG2 cells. As shown in Fig. 4a, co-cultures of HepG2 and CRC cells were treated with 500 μM of CAP for 48 h and concentrations of CAP, 5′-DFCR, 5′-DFUR and 5-FU in five CRC cell lines were determined using the established LC–MS/MS method. Compared to monolayer CRC cells, the concentration of CAP were significantly decreased, and concentrations of 5′-DFCR and 5′-DFUR were moderately increased in the co-culture system. In particular, the concentration of 5-FU in CRC cells was significantly increased by approximately 3–10 times with metabolic activation in co-cultures, suggesting that CAP was effectively metabolized to its active metabolite to exert anti-tumor activity, which was consistent with the results in model of pre-metabolism by hepatocytes (Fig. 6b–f). In addition to 5′-DFCR, 5′-DFUR and 5-FU, secondary metabolites of 5-FU were determined. Generally, 5-FU undergoes either catabolism by dihydropyrimidine dehydrogenase (DPD) or anabolism by thymidine phosphorylase (TP). The former enzyme is responsible for detoxification and subsequent elimination, which ultimately forms α-fluoro-β-alanine (FBAL) without anti-neoplastic effect. The anabolic process results in the formation of three compounds responsible for anti-proliferative activity, namely FUTP, FdUTP, and FdUMP [25] (Fig. 6a). As the cytotoxicity of CAP or 5-FU depends on three compounds, it would be necessary to monitor their intracellular concentrations. Generally, concentrations of FUTP, FdUTP and FdUMP detected by the semi-quantitative method were elevated in co-cultures of CRC cells with HepG2 cells. These results showed that concentrations of CAP and its metabolites in the five CRC cell lines were significantly increased when co-cultured with HepG2 cells, suggesting that the co-culture system could meet the requirements for PK studies of liver-activated CAP-like prodrugs in vitro.

### PK-PD correlation analysis of CAP in CRC cell lines

**Fig. 7:**
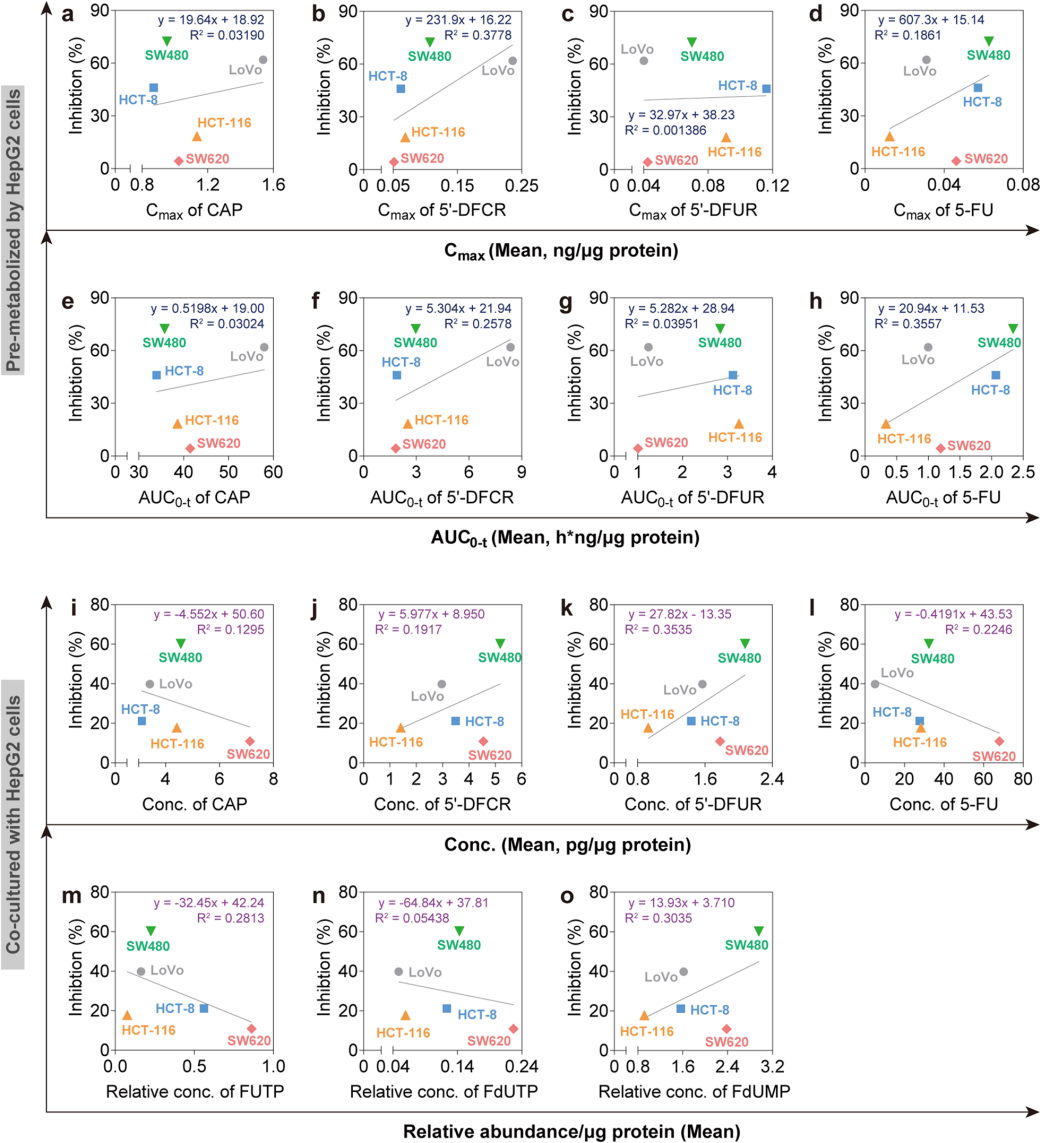
Correlation analysis between pharmacokinetics and relative inhibition among CRC cells. Correlation analysis was conducted between C_max_ (**a**–**d**) or AUC_0-t_ (**e**–**h**) of CAP, 5′-DFCR, 5′-DFUR or 5-FU and the relative inhibition among CRC cells under pre-metabolized by HepG2 cells, respectively. Correlation analysis was conducted between intracellular concentrations of CAP, 5′-DFCR, 5′-DFUR or 5-FU (**i**–**l**) or relative concentrations of FUTP, FdUTP or FdUMP (**m**–**o**) and the relative inhibition among CRC cells under co-cultured with HepG2 cells, respectively

To further investigate the PK and PD relevance of CAP, concentration-effect curves (C-E curves) of CAP in the five CRC cell lines were plotted using the two models of pre-metabolism by HepG2 cells and a co-culture system. Under pre-metabolized by HepG2 cells for 48 h, C_max_ (Fig. 7a–d) or AUC_0-t_ (Fig. 7e–h) of each analyte (CAP and its metabolites) in each CRC cell lines was used as the x-axis, and the corresponding cytotoxicity was used as the y-axis. Each linear regression equation was conducted and the correlation coefficient (R^2^) was used for the degree of the curve fit. The inhibitory effect and PK parameters (C_max_ or AUC_0-t_) of CAP and its metabolites appeared a non-linear relationship. In the co-culture system, the concentration of each analyte (CAP and its metabolites) in each CRC cell line was used as the x-axis, and the corresponding apoptosis rate was used as the y-axis. Linear fitting curves were conducted and the correlation coefficient (R^2^) indicated the degree of curve fit (Fig. 7i–o). The results revealed that the apoptosis rate had a nonlinear relationship with concentrations of CAP and its metabolites. PK-PD correlation analysis indicated that PK behaviors of CAP and its metabolites did not linearly correlate with their differential efficacy on the five CRC cell lines.

## Discussion

CAP, an oral tumor-selective prodrug of 5-FU, has been approved by the FDA as first-line therapy for patients with metastatic colorectal cancer (mCRC). The PK behavior of CAP is characterized by rapid and almost complete gastrointestinal absorption, followed by three metabolic steps that produce 5-FU (Fig. 1a). The expression of TP is significantly higher and the expression of DPD is lower in tumor cells than in normal cells, which contributes to the tumor-specific conversion and targeted intra-tumoral cytotoxicity of CAP [26–28]. Given this tumor-targeting performance, CAP has been demonstrated to produce a lower incidence of serious adverse events (such as neutropenia, nausea, and emesis) than intravenous 5-FU based chemotherapy regimens, although hand-foot syndrome (HFS) and hyperbilirubinemia are more frequently observed with CAP treatment [29, 30].

However, both clinical CAP and 5-FU administration are based on BSA, which has proven to be an imprecise method for determining the optimal dose for a patient. Only 20–30% of patients receiving intravenous 5-FU achieved plasma 5FU concentrations in the appropriate therapeutic range, approximately 40–60% of patients were underdosed and 10–20% overdosed. The 5-FU tissue concentrations were at least 10 times higher than the plasma concentrations after 5-FU treatment based on BSA within 48 h, and 5-FU was retained for a much more extended time in tissues than in plasma [26, 30, 31]. Moreover, patients with CRC who experienced skeletal muscle mass (SMM) loss during CAP treatment were at an increased risk of developing severe toxicity and had shorter survival time [32]. Nevertheless, no changes in the PK properties of CAP and 5-FU were observed in patients with a low SMM. Therefore, the previously identified increased toxicity and shorter survival in patients with a low SMM, could not be explained by alterations in PK characteristics of CAP and its metabolites [33]. Taken together, substantial evidence indicated that systemic exposure to CAP and its metabolites in plasma was poorly predictive of safety and efficacy, which seemed to defeat the value of therapeutic drug monitoring for dosage adjustment [15]. A growing number of similar studies have found that classical PK studies based on plasma drug concentrations cannot fully explain the pharmacological effects of drugs in specific tissues (e.g., tumor, brain), and it is often tricky to truly describe PK profiles and effectively predict the toxicity and efficacy of drugs in vivo, accompanied by the irrelevance of PK/PD [34, 35].

In the present study, PK-PD correlation analysis indicated that PK behaviors of CAP and its metabolites did not linearly correlate with their differential efficacy on five CRC cell lines. In general, the degradation caused by oxidation, defluorination, hydrogenation, hydrolysis, and bond cleavage might be the key factors. When employing a combination of medication, antagonist, and chemotherapy effects might be the probable reason for the nonlinear growth inhibitory effect [36]. Chemotherapy resistance and individual differences are unavoidable challenges in CRC treatment, which are closely related to the tumor immune microenvironment. Deng et al. demonstrated that fucoidan had great potential to be used in tumor immunotherapy, especially when combined with CAP. Fucoidan promoted M1 macrophage differentiation and enhanced the chemotherapeutic sensitivity of CAP on CRC [37]. It suggested that tumor-microenvironment played an important role in the action and mechanism of CAP, which requested further explorations. As a matter of fact, at least 1/3 of the drug targets are located inside the cells, such as DNA, nuclear receptors, various kinases, metabolic enzymes, etc. Representative drugs include anti-biotics (azithromycin and moxifloxacin), anti-malarials (chloroquine), and anti-cancer drugs (adriamycin, paclitaxel, and 5-FU) [38]. For drugs with intracellular targets, such as CAP and 5-FU, it is more critical to explore drug concentrations in cells/subcellular organelles over time than in plasma. It has been evidenced that precise dosing methods, such as DPD enzyme activity testing and, in the case of intravenous 5-FU, PK guided dosing, could reduce toxicity and produce better patient prognosis [30].

Co-culture systems are the basis for any cell–cell interaction exploration. For certain cell populations, the presence of another cell population may improve culturing success or cell behavior to exhibit desired in vivo physiological behaviors [21, 39, 40]. Accordingly, we established two models of CRC cells with the intervention of hepatocytes for the first time, namely pre-metabolism by hepatocytes and co-culture with hepatocytes (Figs. 3, 4a), in which CAP could be metabolized to active metabolites. We observed differential efficacy of CAP in five different CRC cell lines, consistent with the distinct differences in the individual clinical susceptibility to CAP administration, due to primary and acquired resistance [9]. In accordance with the differential efficacy of 5-FU in monolayer CRC cells (Fig. 2b–f), SW480 and LoVo cells were highly sensitive, HCT-8 and HCT-116 cells were intermediately sensitive, and SW620 cells were resistant to CAP treatment in the two models (Figs. 3, 4). Several ABC transporters seem to play a role in fluoropyrimidine-based chemotherapeutic response [41]. The genetic variant of the *ABCB1* (P-gp) transporter gene has been correlated with treatment outcomes and/or occurrence of toxicity [42]. 5-FU and its metabolites have been identified as substrates transported by other ABC family members, such as *ABCC3*, *ABCC4*, *ABCC5*, and *ABCG2* [43, 44]. Thus, *ABCB1* polymorphisms are associated with fluoropyrimidine-related adverse effects, suggesting that P-gp (*ABCB1*) may be involved in the transport of CAP or its metabolites. However, the relationship between P-gp (*ABCB1*), and CAP and its metabolites remains under investigation. Moreover, the PK properties of CAP were successfully evaluated in the two established models. Exposures to metabolites of CAP were dramatically improved in the two models, which were almost absent with the absence of hepatocytes. However, we observed that the PK profile of CAP itself was slightly altered in the five CRC cell lines under pre-metabolized by HepG2 cells (Fig. 5a–e). In contrast, concentrations of CAP were decreased under co-cultured with HepG2 cells (Fig. 6b–f). As one of the enzymes responsible for metabolizing CAP, CyD was present both in the liver and tumor, and the action of CyD only lay in HepG2 cells under pre-metabolized by HepG2 cells. In contrast, the act of CyD existed both in HepG2 and CRC cells in co-cultures, suggesting that CAP was more adequately metabolized in co-culture systems. However, it could be seen as a limitation of our study that co-cultures consisting of one kind of liver cell line and one kind of intestinal cell line were far from organoids, and it was challenging to fully simulate the real in vivo environment. For example, Kimura et al*.* [45] confirmed that PK and PD properties of anti-tumor drugs could be investigated via a simple microfluidic device that used Caco-2, HepG2, and A549 cell cultures as organ models.

As with other cytotoxic drugs, the interpatient variability of the PK parameters of CAP and its metabolites is high (27 to 89%) and is most likely to be primarily due to variability in the activity of the various enzymes involved in the metabolism of CAP and its metabolites. High interpatient variability in CES, CyD, and DPD activities within a tumor type has been reported [46, 47]. Because plasma concentrations of 5-FU do not reflect tissue concentrations of 5-FU following CAP administration and there is no close relationship between the pharmacokinetics of CAP and its safety and efficacy [14, 15], this variability should not be of clinical concern. Preclinical results suggested that the ratio of TP to DPD determined whether an anti-tumor agent had a cytotoxic effect on the tumor or not [48]. Moreover, the inhibition of DPD has been demonstrated to enhance intra-tumoral 5-FU concentration, which has been tested with several agents that are under development (e.g. 5-ethynyl-uracil) [30, 49]. To discuss whether genetic variants of related metabolic enzymes affect the pharmacokinetic profiles of CAP, we used three enzyme inhibitors to co-treat with CAP in co-culture systems (Additional file 1: Fig. S5).

By using CAP as a model drug, the establishment of the co-culture model provided insight into the cellular PK and PD evaluation of CAP-like prodrugs in vitro, and was ultimately beneficial for optimizing current treatment regimens. Furthermore, it supplied a model and method for preclinical studies on prodrugs that require hepatic activation in the development of new drugs. In general, such prodrugs were difficult to be screened rapidly by simple cell models in vitro and could only be examined by preclinical animal models for efficacy and PK behaviors. The robust, reproducible, simple and cost‑effective models offered the possibility of accelerating the drug development process and saving research and development (R&D) costs, and filling the gap for rapid screening of such new prodrugs. Subsequently, candidate compounds screened by this in vitro model could be further evaluated by animal models.

## Conclusion

**Fig. 8:**
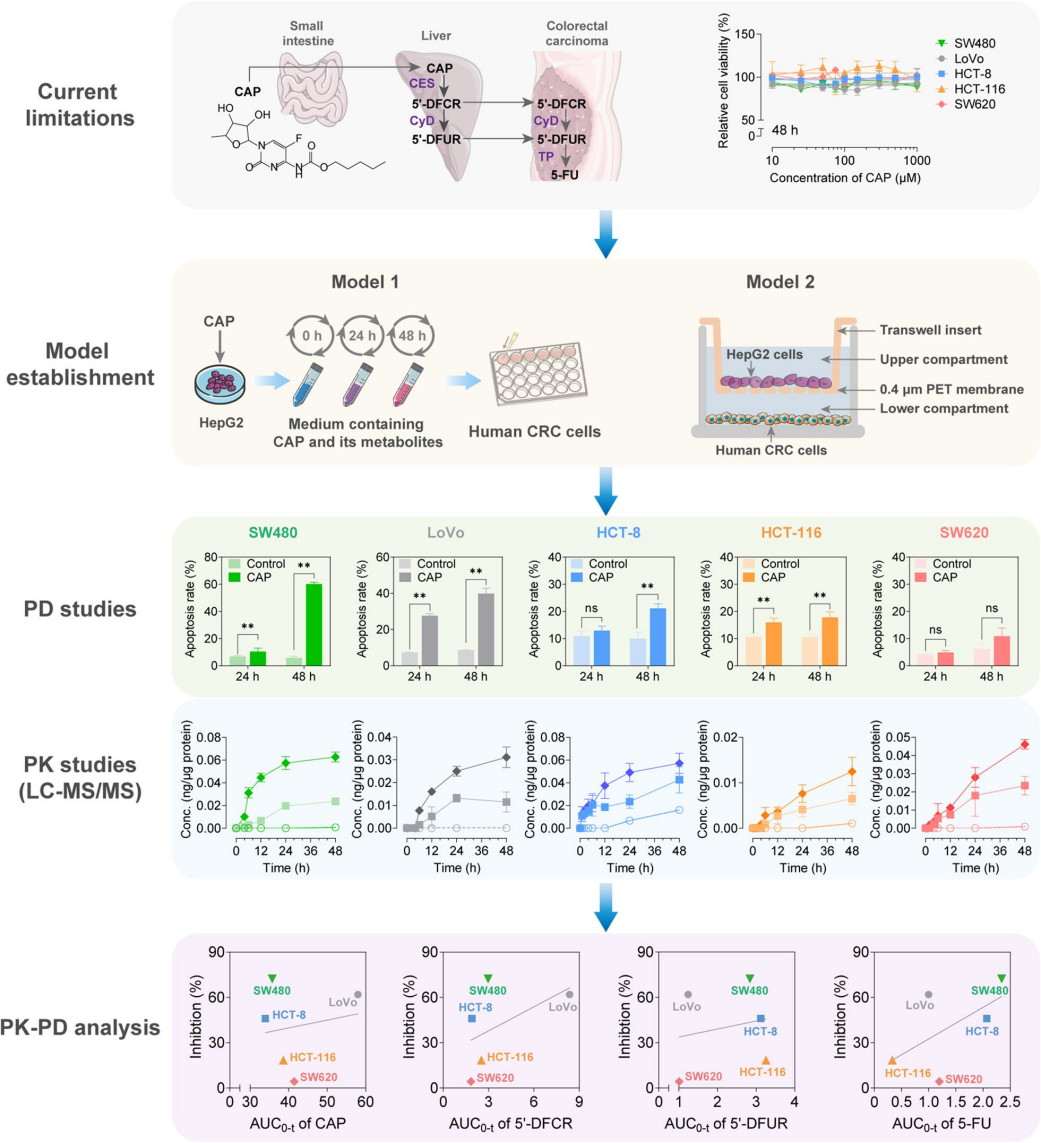
Schematic diagram of for cellular PK and PD studies of CAP in co-culture systems in vitro

In summary, we established in vitro co-culture cellular models of hepatic and intestinal cells and successfully applied them to PK and PD studies of the prodrug, CAP. Compared with monolayer cells, CAP exerted significantly enhanced anti-tumor activities on CRC cell lines in established models, and it appeared differential efficacy on five CRC cell lines. Moreover, exposures to metabolites of CAP were dramatically elevated in the two models, whereas these metabolites were almost absent in monolayer cell lines, suggesting that the established models could mimic the process of metabolic conversion from CAP to active metabolites. In addition, PK-PD correlation analysis indicated no relevant relationships between PK behaviors of CAP and its metabolites and their differential efficacy on the five CRC cell lines (R^2^ < 0.5). To our knowledge, it was the first time to investigate cellular PK and PD characteristics of CAP and its metabolites in vitro, showing that PK and PD properties of CAP were remarkably improved by hepatocytes intervention. Our established models involving hepatocytes intervention could allow the PK and PD studies of prodrugs in vitro that need to be metabolically activated (Fig. 8).

## Supplementary Information

**Additional file 1**: **Fig. S1** Chemical structural of CAP (**a**), 5′-DFCR (**b**), 5′-DFUR (**c**), 5-FU (**d**), FUTP (**e**), FdUTP (**f**), FdUMP (**g**) and tolbutamide (IS) (**h**). **Fig. S2 **One-way ANOVA test on the drug sensitivities of CAP (a) and 5-FU (b and c). **Fig. S3** The comparation of metabolic capability between HepG2 cells and mouse primary hepatocytes, indicating by concentrations of 5'-DFCR (**a**), 5'-DFUR (**b**), and 5-FU (**c**) in the medium. **Fig. S4** Quantification of the western blot band intensity was performed using ImageJ and GAPDH was used as loading controls. Data were expressed as the mean ± SD, and the results represented three independent experiments. ^*^P < 0.05, ^**^P < 0.01 vs co-culture (-) CAP 0 μM, ^#^*P* < 0.05, ^##^*P* < 0.01 vs co-culture (-) CAP 500 μM. **Fig. S5 **Cellular pharmacokinetic profiles of CAP, 5′-DFCR, 5′-DFUR and 5-FU in CRC cells after treatment with CAP and related enzyme inhibitors (WWL113, an inhibitor of CES; tetrahydrouridine, an inhibitor of CyD; gimeracil, an inhibitor of DPD) under co-cultured with HepG2 cells. **Table S1** Pharmacokinetic parameters of 5'-DFCR, 5'-DFUR and 5-FU in the medium of HepG2 cells and mouse primary hepatocytes cells after CAP administration (500 μM) (Mean ± SD, n = 4).

**Additional file 2**: Methodology validation of CAP, 5′-DFCR, 5′-DFUR and 5-FU. **Fig. S6** Ion transitions from parent ion to daughter ion of CAP (**a**), 5′-DFCR (**b**), 5′-DFUR (**c**), 5-FU (**d**) and tolbutamide (IS) (**e**). **Fig. S7** Representative chromatograms of CAP, 5′-DFCR, 5′-DFUR, 5-FU and tolbutamide (IS). **a** Blank matrix. **b**–**f** Blank matrix spiked with 150 ng/mL of CAP, 5′-DFCR, 5′-DFUR, 5-FU and 200 ng/mL of tolbutamide (IS). **g**–**k** CAP, 5′-DFCR, 5′-DFUR, 5-FU and tolbutamide (IS) in lysate sample of LoVo cells under co-cultured with HepG2 cells for 48 h. **Fig S8** Calibration curves for CAP (**a**), 5′-DFCR (**b**), 5′-DFUR (**c**), 5-FU (**d**). **Table S2** Calibration curves of CAP, 5′-DFCR, 5′-DFUR and 5-FU (n = 6). **Table S3** Intra-assay and inter-assay accuracy and precision of CAP, 5′-DFCR, 5′-DFUR and 5-FU (n = 6). **Table S4** Assessment of the matrix effect and recovery of CAP, 5′-DFCR, 5′-DFUR and 5-FU (n = 6). **Table S5** Stability results of CAP, 5′-DFCR, 5′-DFUR and 5-FU (n = 3). **Table S6** Dilution integrity of CAP, 5′-DFCR, 5′-DFUR and 5-FU (n = 6).

**Additional file 3**: One-way ANOVA test on the drug sensitivities of CAP and 5-FU.

**Additional file 4**: LC-MS/MS raw data.

## Data Availability

The analyzed data during the current study are available from the corresponding author on reasonable request.

## Abbreviations

5′-DFCR: 5′-Deoxy-5-fluorocytidine
5′-DFUR: 5′-Deoxy-5-fluorouridine
5-FU: 5-Fluorouracil
BCA: Bicinchoninic acid
BSA: Body surface area
CAP: Capecitabine
CCK-8: Cell Counting Kit-8
CES: Carboxylesterase
CRC: Colorectal cancer
CyD: Cytidine deaminase
DPD: Dihydropyrimidine dehydrogenase
ECL: Enhanced chemiluminescence
FBAL: α-Fluoro-β-alanine
FBS: Fetal bovine serum
FDA: Food and Drug Administration
FdUMP: 5-Fluoro-2′-deoxyuridine 5′-monophosphate
FdUTP: 5-Fluoro-2′-deoxyuridine 5′-triphosphate
FITC: Fluorescein isothiocyanate
FUTP: 5-Fluorouridine 5′-triphosphate
HFS: Hand-foot syndrome
HPLC: High-performance liquid chromatography
HRP: Horseradish peroxidase
HUVEC: Human umbilical vein EC cell
IS: Internal standard
LC–MS/MS: Liquid chromatography–tandem mass spectrometry
MRM: Multiple-reaction monitoring
PD: Pharmacodynamic
PI: Propidium iodide
PK: Pharmacokinetic
R&D: Research and development
SD: Standard deviation
SMM: Skeletal muscle mass
TP: Thymidine phosphorylase

## References

[1] Siegel RL, Miller KD, Goding Sauer A, Fedewa SA, Butterly LF, Anderson JC (2020). Colorectal cancer statistics, 2020. CA Cancer J Clin.

[2] Punt CJ, Koopman M, Vermeulen L (2017). From tumour heterogeneity to advances in precision treatment of colorectal cancer. Nat Rev Clin Oncol.

[3] van der Geest LG, Lam-Boer J, Koopman M, Verhoef C, Elferink MA, de Wilt JH (2015). Nationwide trends in incidence, treatment and survival of colorectal cancer patients with synchronous metastases. Clin Exp Metastasis.

[4] Lieberman D, Ladabaum U, Cruz-Correa M, Ginsburg C, Inadomi JM, Kim LS (2016). Screening for colorectal cancer and evolving issues for physicians and patients: a review. JAMA.

[5] Vogel A, Hofheinz RD, Kubicka S, Arnold D (2017). Treatment decisions in metastatic colorectal cancer—beyond first and second line combination therapies. Cancer Treat Rev.

[6] Van der Jeught K, Xu HC, Li YJ, Lu XB, Ji G (2018). Drug resistance and new therapies in colorectal cancer. World J Gastroenterol.

[7] Benson ABVA, Cederquist L, Chan E, Chen YJ, Cooper HS, Deming D, Engstrom PF, Enzinger PC, Fichera A, Grem JL, Grothey A, Hochster HS, Hoffe S, Hunt S, Kamel A, Kirilcuk N, Krishnamurthi S, Messersmith WA, Mulcahy MF, Murphy JD, Nurkin S, Saltz L, Sharma S, Shibata D, Skibber JM, Sofocleous CT, Stoffel EM, Stotsky-Himelfarb E, Willett CG, Wu CS, Gregory KM, Freedman-Cass D (2017). Colon cancer, version 12017, NCCN clinical practice guidelines in oncology. J Natl Compr Canc Netw.

[8] Kelly C, Cassidy J (2007). Capecitabine in the treatment of colorectal cancer. Expert Rev Anticancer Ther.

[9] Crea F, Nobili S, Paolicchi E, Perrone G, Napoli C, Landini I (2011). Epigenetics and chemoresistance in colorectal cancer: an opportunity for treatment tailoring and novel therapeutic strategies. Drug Resist Updates.

[10] Alvarez P, Marchal JA, Boulaiz H, Carrillo E, Velez C, Rodriguez-Serrano F (2012). 5-Fluorouracil derivatives: a patent review. Expert Opin Ther Pat.

[11] Van Cutsem E, Twelves C, Cassidy J, Allman D, Bajetta E, Boyer M (2001). Oral capecitabine compared with intravenous fluorouracil plus leucovorin in patients with metastatic colorectal cancer: results of a large phase III study. J Clin Oncol.

[12] Hoff PM, Ansari R, Batist G, Cox J, Kocha W, Kuperminc M (2001). Comparison of oral capecitabine versus intravenous fluorouracil plus leucovorin as first-line treatment in 605 patients with metastatic colorectal cancer: results of a randomized phase III study. J Clin Oncol.

[13] Lee JJ, Beumer JH, Chu E (2016). Therapeutic drug monitoring of 5-fluorouracil. Cancer Chemother Pharmacol.

[14] Urien S, Rezai K, Lokiec F (2005). Pharmacokinetic modelling of 5-FU production from capecitabine–a population study in 40 adult patients with metastatic cancer. J Pharmacokinet Pharmacodyn.

[15] Gieschke R, Burger HU, Reigner B, Blesch KS, Steimer JL (2003). Population pharmacokinetics and concentration-effect relationships of capecitabine metabolites in colorectal cancer patients. Br J Clin Pharmacol.

[16] Tiede LM, Cook EA, Morsey B, Fox HS (2011). Oxygen matters: tissue culture oxygen levels affect mitochondrial function and structure as well as responses to HIV viroproteins. Cell Death Dis.

[17] Redshaw Z, Loughna PT (2012). Oxygen concentration modulates the differentiation of muscle stem cells toward myogenic and adipogenic fates. Differentiation.

[18] Kuriwaka M, Ochi M, Uchio Y, Maniwa S, Adachi N, Mori R (2003). Optimum combination of monolayer and three-dimensional cultures for cartilage-like tissue engineering. Tissue Eng.

[19] Shimasaki T, Yamamoto S, Arisawa T (2018). Exosome research and co-culture study. Biol Pharm Bull.

[20] Wu MH, Huang SB, Lee GB (2010). Microfluidic cell culture systems for drug research. Lab Chip.

[21] Goers L, Freemont P, Polizzi KM (2014). Co-culture systems and technologies: taking synthetic biology to the next level. J Royal Soc Interface.

[22] Guzzardi MA, Vozzi F, Ahluwalia AD (2009). Study of the crosstalk between hepatocytes and endothelial cells using a novel multicompartmental bioreactor: a comparison between connected cultures and cocultures. Tissue Eng Part A.

[23] Choe A, Ha SK, Choi I, Choi N, Sung JH (2017). Microfluidic Gut-liver chip for reproducing the first pass metabolism. Biomed Microdevice.

[24] Low YL, Pan Y, Short JL, Nicolazzo JA (2020). Development and validation of a LC-MS/MS assay for quantifying the uptake of docosahexaenoic acid-d5 into mouse microglia. J Pharm Biomed Anal.

[25] Longley DB, Harkin DP, Johnston PG (2003). 5-fluorouracil: mechanisms of action and clinical strategies. Nat Rev Cancer.

[26] Schüller J, Cassidy J, Dumont E, Roos B, Durston S, Banken L (2000). Preferential activation of capecitabine in tumor following oral administration to colorectal cancer patients. Cancer Chemother Pharmacol.

[27] Bonotto M, Bozza C, Di Loreto C, Osa EO, Poletto E, Puglisi F (2013). Making capecitabine targeted therapy for breast cancer: which is the role of thymidine phosphorylase?. Clin Breast Cancer.

[28] Diasio RB, Harris BE (1989). Clinical pharmacology of 5-fluorouracil. Clin Pharmacokinet.

[29] Saif MW, Katirtzoglou NA, Syrigos KN (2008). Capecitabine: an overview of the side effects and their management. Anticancer Drugs.

[30] Schneider JJ, Galettis P, Martin JH (2021). Overcoming barriers to implementing precision dosing with 5-fluorouracil and capecitabine. Br J Clin Pharmacol.

[31] Derissen EJ, Jacobs BA, Huitema AD, Rosing H, Schellens JH, Beijnen JH (2016). Exploring the intracellular pharmacokinetics of the 5-fluorouracil nucleotides during capecitabine treatment. Br J Clin Pharmacol.

[32] Kurk S, Peeters P, Stellato R, Dorresteijn B, de Jong P, Jourdan M (2019). Skeletal muscle mass loss and dose-limiting toxicities in metastatic colorectal cancer patients. J Cachexia Sarcopenia Muscle.

[33] Molenaar-Kuijsten L, Jacobs BAW, Kurk SA, May AM, Dorlo TPC, Beijnen JH (2021). Worse capecitabine treatment outcome in patients with a low skeletal muscle mass is not explained by altered pharmacokinetics. Cancer Med.

[34] Dalhoff A (2014). Pharmacokinetics and pharmacodynamics of aerosolized antibacterial agents in chronically infected cystic fibrosis patients. Clin Microbiol Rev.

[35] Drollmann A, Brown M, Sechaud R, Perry S, Hara H, Jones I (2014). Effect of dual bronchodilation with QVA149 on cardiac safety in healthy volunteers. Int J Clin Pharmacol Ther.

[36] Rakitina TV, Vasilevskaya IA, O'Dwyer PJ (2003). Additive interaction of oxaliplatin and 17-allylamino-17-demethoxygeldanamycin in colon cancer cell lines results from inhibition of nuclear factor kappaB signaling. Can Res.

[37] Deng Z, Wu N, Suo Q, Wang J, Yue Y, Geng L (2022). Fucoidan, as an immunostimulator promotes M1 macrophage differentiation and enhances the chemotherapeutic sensitivity of capecitabine in colon cancer. Int J Biol Macromol.

[38] Zhang J, Zhou F, Lu M, Ji W, Niu F, Zha W (2012). Pharmacokinetics-pharmacology disconnection of herbal medicines and its potential solutions with cellular pharmacokinetic-pharmacodynamic strategy. Curr Drug Metab.

[39] Zengler K, Toledo G, Rappe M, Elkins J, Mathur EJ, Short JM (2002). Cultivating the uncultured. Proc Natl Acad Sci USA.

[40] Larose C, Berger S, Ferrari C, Navarro E, Dommergue A, Schneider D (2010). Microbial sequences retrieved from environmental samples from seasonal arctic snow and meltwater from Svalbard, Norway. Extremophiles.

[41] Nies AT, Magdy T, Schwab M, Zanger UM (2015). Role of ABC transporters in fluoropyrimidine-based chemotherapy response. Adv Cancer Res.

[42] Garcia-Gonzalez X, Cortejoso L, Garcia MI, Garcia-Alfonso P, Robles L, Gravalos C (2015). Variants in CDA and ABCB1 are predictors of capecitabine-related adverse reactions in colorectal cancer. Oncotarget.

[43] Yuan J, Lv H, Peng B, Wang C, Yu Y, He Z (2009). Role of BCRP as a biomarker for predicting resistance to 5-fluorouracil in breast cancer. Cancer Chemother Pharmacol.

[44] Lou Y, Wang Q, Zheng J, Hu H, Liu L, Hong D (2016). Possible pathways of capecitabine-induced hand-foot syndrome. Chem Res Toxicol.

[45] Kimura H, Ikeda T, Nakayama H, Sakai Y, Fujii T (2015). An on-chip small intestine-liver model for pharmacokinetic studies. J Lab Autom.

[46] Miwa M, Ura M, Nishida M, Sawada N, Ishikawa T, Mori K (1998). Design of a novel oral fluoropyrimidine carbamate, capecitabine, which generates 5-fluorouracil selectively in tumours by enzymes concentrated in human liver and cancer tissue. Eur J Cancer.

[47] Guimbaud R, Guichard S, Dusseau C, Bertrand V, Aparicio T, Lochon I (2000). Dihydropyrimidine dehydrogenase activity in normal, inflammatory and tumour tissues of colon and liver in humans. Cancer Chemother Pharmacol.

[48] Ishikawa T, Sekiguchi F, Fukase Y, Sawada N, Ishitsuka H (1998). Positive correlation between the efficacy of capecitabine and doxifluridine and the ratio of thymidine phosphorylase to dihydropyrimidine dehydrogenase activities in tumors in human cancer xenografts. Can Res.

[49] Milano G, McLeod HL (2000). Can dihydropyrimidine dehydrogenase impact 5-fluorouracil-based treatment?. Eur J Cancer.

