# Supplementary material for: In vitro co-culture systems of hepatic and intestinal cells for cellular pharmacokinetic and pharmacodynamic studies of capecitabine against colorectal cancer
Source: Cancer Cell Int. 2023 Jan 31;23:14. doi: 10.1186/s12935-023-02853-6 (PMC9887786; doi:10.1186/s12935-023-02853-6)
Supplement: Supplementary file 1 — Additional file 1: Fig. S1 Chemical structural of CAP (a), 5′-DFCR (b), 5′-DFUR (c), 5-FU (d), FUTP (e), FdUTP (f), FdUMP (g) and tolbutamide (IS) (h). Fig. S2 One-way ANOVA test on the drug sensitivities of CAP (a) and 5-FU (b and c). Fig. S3 The comparation of metabolic capability between HepG2 cells and mouse primary hepatocytes, indicating by concentrations of 5'-DFCR (a), 5'-DFUR (b), and 5-FU (c) in the medium. Fig. S4 Quantification of the western blot band intensity was performed using ImageJ and GAPDH was used as loading controls. Data were expressed as the mean ± SD, and the results represented three independent experiments. *P < 0.05, **P < 0.01 vs co-culture (-) CAP 0 μM, #P < 0.05, ##P < 0.01 vs co-culture (-) CAP 500 μM. Fig. S5 Cellular pharmacokinetic profiles of CAP, 5′-DFCR, 5′-DFUR and 5-FU in CRC cells after treatment with CAP and related enzyme inhibitors (WWL113, an inhibitor of CES; tetrahydrouridine, an inhibitor of CyD; gimeracil, an inhibitor of DPD) under co-cultured with HepG2 cells. Table S1 Pharmacokinetic parameters of 5'-DFCR, 5'-DFUR and 5-FU in the medium of HepG2 cells and mouse primary hepatocytes cells after CAP administration (500 μM) (Mean ± SD, n = 4). [file 12935_2023_2853_MOESM1_ESM.docx]

**In vitro co-culture systems of hepatic and intestinal cells for cellular pharmacokinetic and pharmacodynamic studies of capecitabine against colorectal cancer**

**List of additional file 1**

**Fig. S1** Chemical structural of CAP (**a**), 5′-DFCR (**b**), 5′-DFUR (**c**), 5-FU (**d**), FUTP (**e**), FdUTP (**f**), FdUMP (**g**) and tolbutamide (IS) (**h**).

**Fig. S2** One-way ANOVA test on the drug sensitivities of CAP (**a**) and 5-FU (**b** and **c**).

**Fig. S3** The comparation of metabolic capability between HepG2 cells and mouse primary hepatocytes, indicating by concentrations of 5'-DFCR (**a**), 5'-DFUR (**b**), and 5-FU (**c**) in the medium.

**Fig. S4** Quantification of the western blot band intensity was performed using ImageJ and GAPDH was used as loading controls. Data were expressed as the mean ± SD, and the results represented three independent experiments. ^*^*P* < 0.05, ^**^*P* < 0.01 vs co-culture (-) CAP 0 μM, ^#^*P* < 0.05, ^##^*P* < 0.01 vs co-culture (-) CAP 500 μM.

**Fig. S5** Cellular pharmacokinetic profiles of CAP, 5′-DFCR, 5′-DFUR and 5-FU in CRC cells after treatment with CAP and related enzyme inhibitors (WWL113, an inhibitor of CES; tetrahydrouridine, an inhibitor of CyD; gimeracil, an inhibitor of DPD) under co-cultured with HepG2 cells.

**Table S1** Pharmacokinetic parameters of 5'-DFCR, 5'-DFUR and 5-FU in the medium of HepG2 cells and mouse primary hepatocytes cells after CAP administration (500 μM) (Mean ± SD, n = 4).

**Fig. S1** Chemical structural of CAP (**a**), 5′-DFCR (**b**), 5′-DFUR (**c**), 5-FU (**d**), FUTP (**e**), FdUTP (**f**), FdUMP (**g**) and tolbutamide (IS) (**h**).


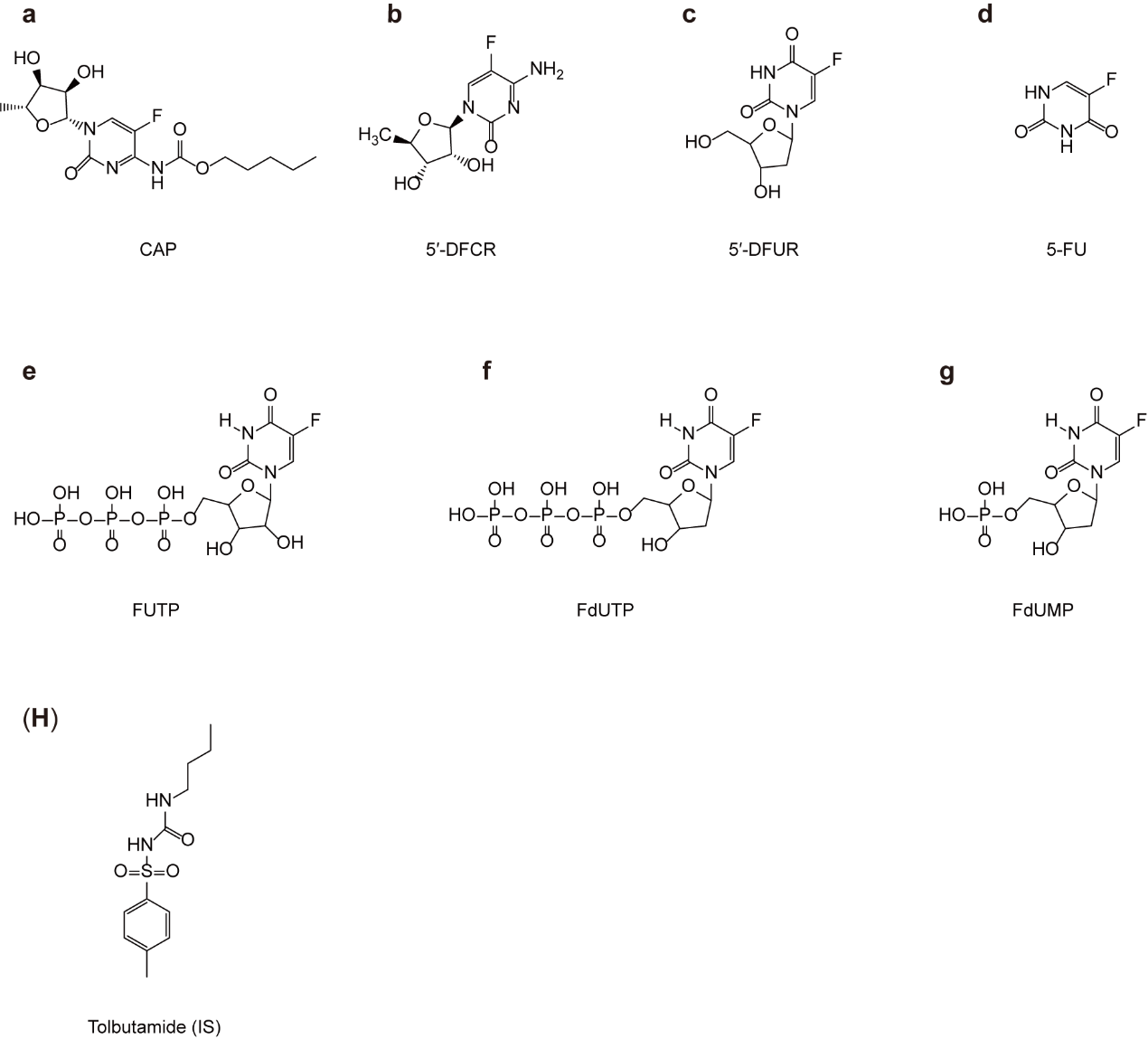


**Fig. S2** One-way ANOVA test on the drug sensitivities of CAP (**a**) and 5-FU (**b** and **c**).


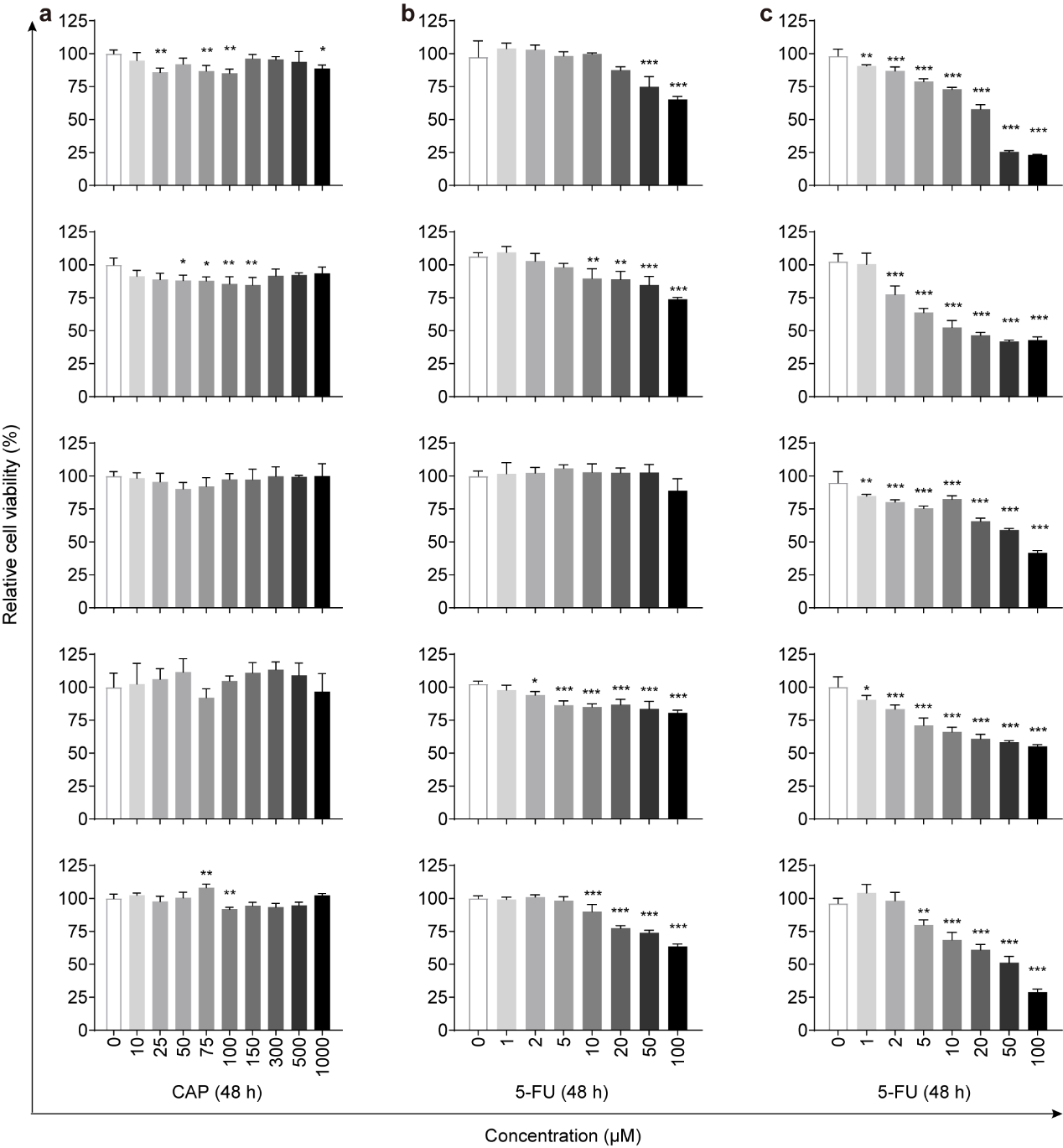


**Fig. S3** The comparation of metabolic capability between HepG2 cells and mouse primary hepatocytes, indicating by concentrations of 5'-DFCR (**a**), 5'-DFUR (**b**), and 5-FU (**c**) in the medium.


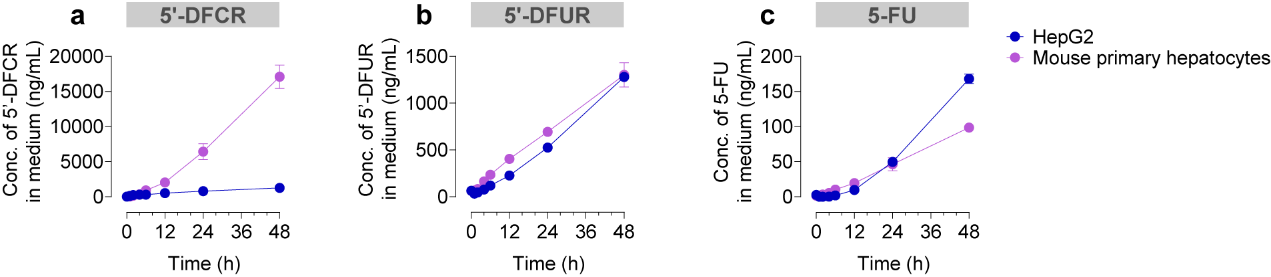


**Fig. S4** Quantification of the western blot band intensity was performed using ImageJ and GAPDH was used as loading controls. Data were expressed as the mean ± SD, and the results represented three independent experiments. ^*^*P* < 0.05, ^**^*P* < 0.01 vs co-culture (-) CAP 0 μM, ^#^*P* < 0.05, ^##^*P* < 0.01 vs co-culture (-) CAP 500 μM.


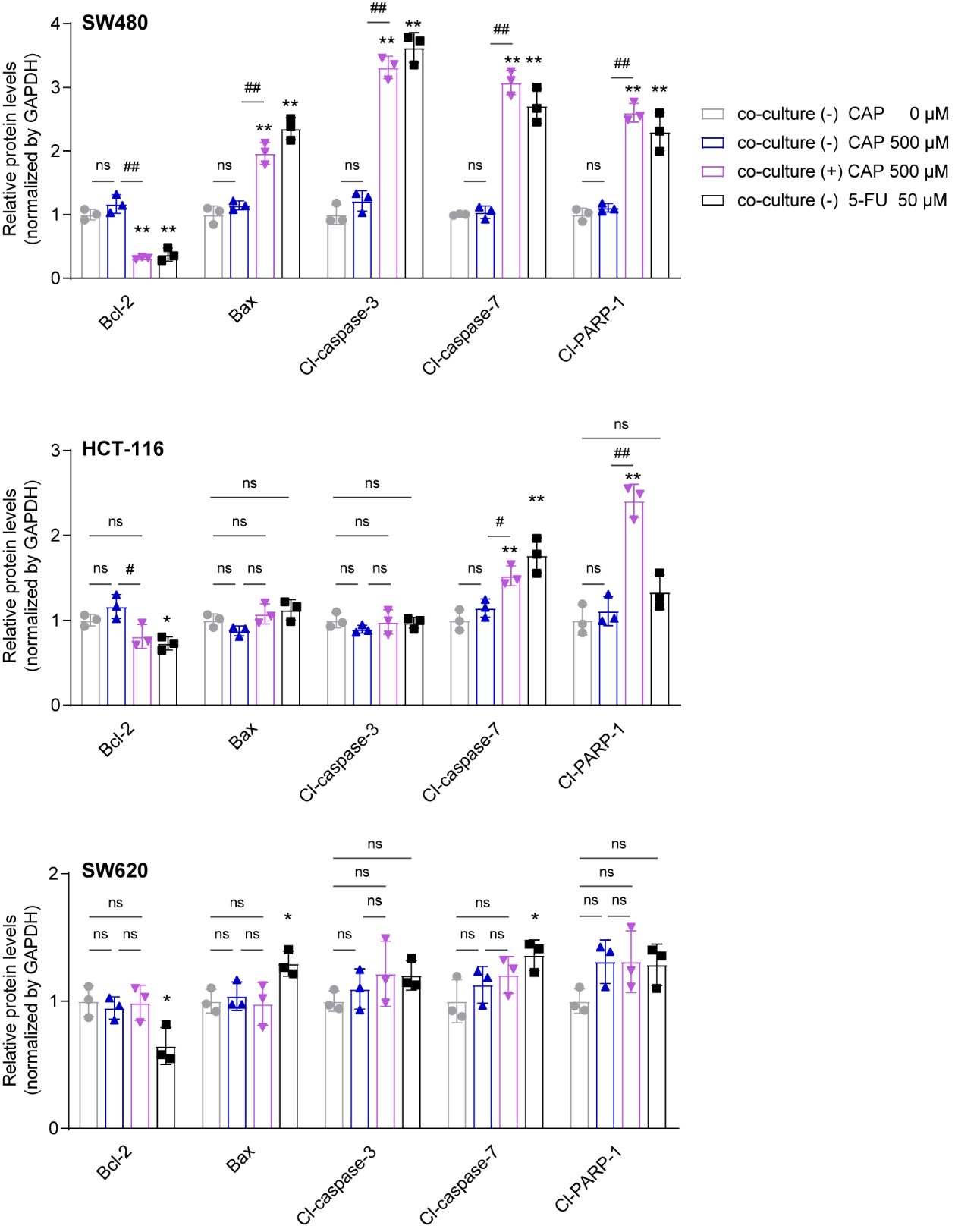


**Fig. S5** Cellular pharmacokinetic profiles of CAP, 5′-DFCR, 5′-DFUR and 5-FU in CRC cells after treatment with CAP and related enzyme inhibitors (WWL113, an inhibitor of CES; tetrahydrouridine, an inhibitor of CyD; gimeracil, an inhibitor of DPD) under co-cultured with HepG2 cells.


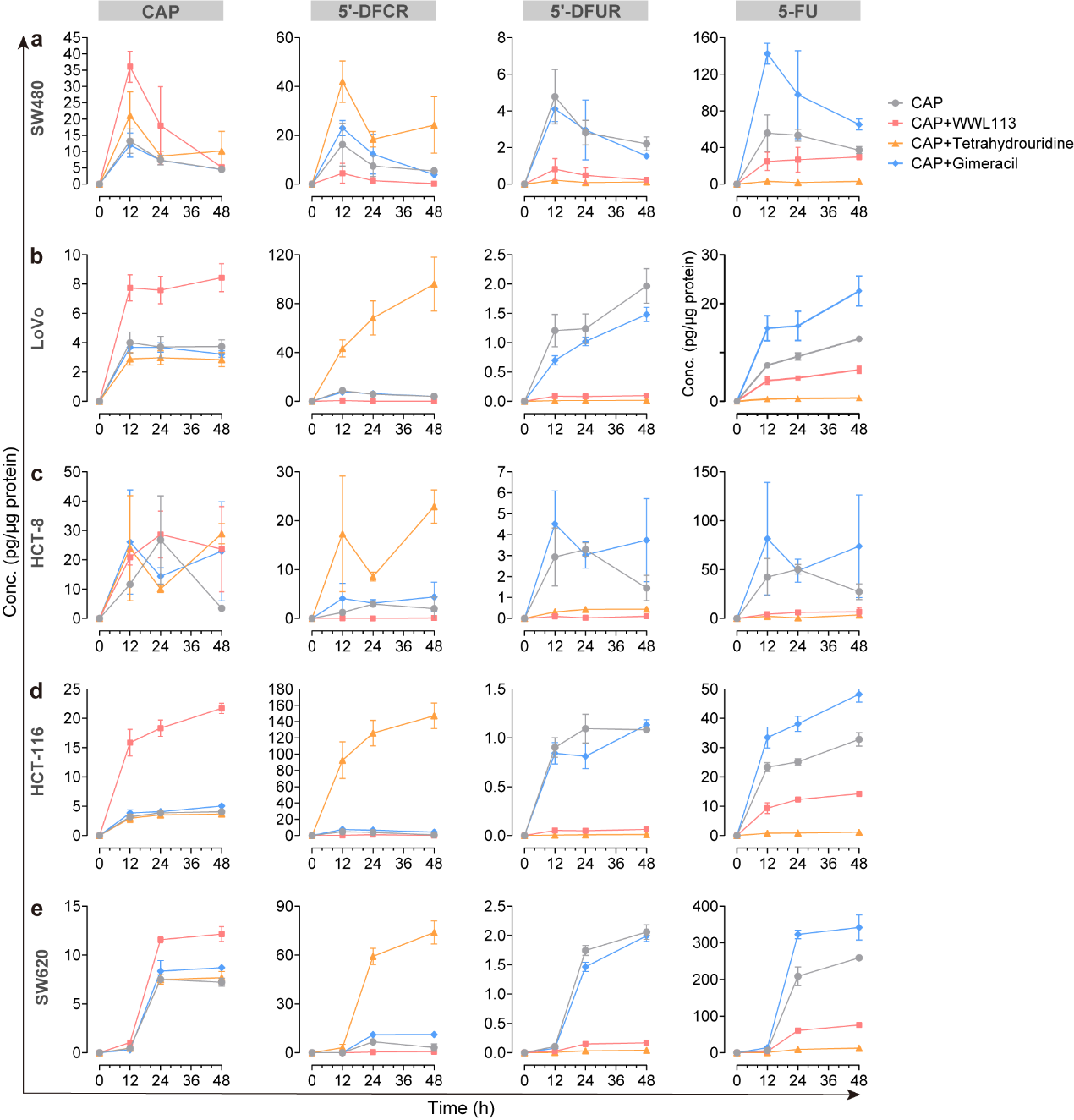


**Table S1** Pharmacokinetic parameters of 5'-DFCR, 5'-DFUR and 5-FU in the medium of HepG2 cells and mouse primary hepatocytes cells after CAP administration (500 μM) (Mean ± SD, n = 4).

| Parameters  (units) | HepG2 cells | | |  | Mouse primary hepatocytes | | |  |
| --- | --- | --- | --- | --- | --- | --- | --- | --- |
|  | 5'-DFCR | 5'-DFUR | 5-FU |  | 5'-DFCR | 5'-DFUR | 5-FU | |
| T_max_ (h) | 48 ± 0 | 48 ± 0 | 48 ± 0 |  | 48 ± 0 | 48 ± 0 | 48 ± 0 | |
| C_max_  (μg/mL) | 1.27 ± 0.048 | 1.28 ± 0.044 | 0.17 ± 0.007 |  | 17.10 ± 1.68^**^ | 1.30 ± 0.13 | 0.10 ± 0.005^**^ | |
| AUC_0-t_ (h*μg/mL) | 36.87 ± 1.66 | 27.64 ± 0.94 | 3.01 ± 0.12 |  | 344.11± 22.76^**^ | 28.13 ±2.07 | 2.25 ± 0.21^**^ | |

T_max_, time to peak concentration; C_max_, peak concentration; AUC_0-t_, area under the concentration-time curve from zero to the time of last measurable concentration.

^*^*P* < 0.05, ^**^*P* < 0.01 compared with HepG2 cells.
