# Supplementary material for: In vitro co-culture systems of hepatic and intestinal cells for cellular pharmacokinetic and pharmacodynamic studies of capecitabine against colorectal cancer
Source: Cancer Cell Int. 2023 Jan 31;23:14. doi: 10.1186/s12935-023-02853-6 (PMC9887786; doi:10.1186/s12935-023-02853-6)
Supplement: Supplementary file 2 — Additional file 2: Methodology validation of CAP, 5′-DFCR, 5′-DFUR and 5-FU. Fig. S6 Ion transitions from parent ion to daughter ion of CAP (a), 5′-DFCR (b), 5′-DFUR (c), 5-FU (d) and tolbutamide (IS) (e). Fig. S7 Representative chromatograms of CAP, 5′-DFCR, 5′-DFUR, 5-FU and tolbutamide (IS). a Blank matrix. b–f Blank matrix spiked with 150 ng/mL of CAP, 5′-DFCR, 5′-DFUR, 5-FU and 200 ng/mL of tolbutamide (IS). g–k CAP, 5′-DFCR, 5′-DFUR, 5-FU and tolbutamide (IS) in lysate sample of LoVo cells under co-cultured with HepG2 cells for 48 h. Fig S8 Calibration curves for CAP (a), 5′-DFCR (b), 5′-DFUR (c), 5-FU (d). Table S2 Calibration curves of CAP, 5′-DFCR, 5′-DFUR and 5-FU (n = 6). Table S3 Intra-assay and inter-assay accuracy and precision of CAP, 5′-DFCR, 5′-DFUR and 5-FU (n = 6). Table S4 Assessment of the matrix effect and recovery of CAP, 5′-DFCR, 5′-DFUR and 5-FU (n = 6). Table S5 Stability results of CAP, 5′-DFCR, 5′-DFUR and 5-FU (n = 3). Table S6 Dilution integrity of CAP, 5′-DFCR, 5′-DFUR and 5-FU (n = 6). [file 12935_2023_2853_MOESM2_ESM.docx]

**In vitro co-culture systems of hepatic and intestinal cells for cellular pharmacokinetic and pharmacodynamic studies of capecitabine against colorectal cancer**

**List of additional file 2**

**Methodology validation of CAP, 5′-DFCR, 5′-DFUR and 5-FU**

**Fig. S6** Ion transitions from parent ion to daughter ion of CAP (**a**), 5′-DFCR (**b**), 5′-DFUR (**c**), 5-FU (**d**) and tolbutamide (IS) (**e**).

**Fig. S7** Representative chromatograms of CAP, 5′-DFCR, 5′-DFUR, 5-FU and tolbutamide (IS). **a** Blank matrix. **b**–**f** Blank matrix spiked with 150 ng/mL of CAP, 5′-DFCR, 5′-DFUR, 5-FU and 200 ng/mL of tolbutamide (IS). **g**–**k** CAP, 5′-DFCR, 5′-DFUR, 5-FU and tolbutamide (IS) in lysate sample of LoVo cells under co-cultured with HepG2 cells for 48 h.

**Fig S8** Calibration curves for CAP (**a**), 5′-DFCR (**b**), 5′-DFUR (**c**), 5-FU (**d**).

**Table S2** Calibration curves of CAP, 5′-DFCR, 5′-DFUR and 5-FU (n = 6).

**Table S3** Intra-assay and inter-assay accuracy and precision of CAP, 5′-DFCR, 5′-DFUR and 5-FU (n = 6).

**Table S4** Assessment of the matrix effect and recovery of CAP, 5′-DFCR, 5′-DFUR and 5-FU (n = 6).

**Table S5** Stability results of CAP, 5′-DFCR, 5′-DFUR and 5-FU (n = 3).

**Table S6** Dilution integrity of CAP, 5′-DFCR, 5′-DFUR and 5-FU (n = 6).

**Methodology validation of CAP, 5′-DFCR, 5′-DFUR and 5-FU**

**1. Method**

**1. Preparation of standard solutions, calibration standards and quality control (QC) samples**

The standard stock solution of CAP or 5′-DFCR was dissolved in methanol, and 5′-DFCR, 5-FU, tolbutamide (IS) were dissolved in double distilled water, 50% methanol, acetonitrile, respectively. Individual standard stock solution (1 mg/mL) and tolbutamide (IS) stock solution (1 mg/mL) were prepared and stored at 4 °C. Using mixed stock solutions of CAP, 5′-DFCR, 5′-DFUR and 5-FU, the working solutions of CAP and its metabolites were obtained by serial diluted with 50% methanol.

Calibration standards were prepared by spiking 90 μL of blank matrix with 10 μL of mixed working solution of CAP and its metabolites at different concentrations to achieve standards with concentrations of 1, 2, 5, 10, 20, 50, 100, 200, 500 and 1000 ng/mL. And 400 μL of de-protein solvent containing 200 ng/mL of tolbutamide (IS) was added in each sample, further processed identically to the *Sample preparation*.

Quality control (QC) samples were prepared by spiking 90 μL of blank matrix with 10 μL of freshly prepared working solutions of 10, 25, 1500 and 8000 ng/mL to obtain the lower limit of quantitation (LLOQ, 1 ng/mL), low quality control (LQC, 2.5 ng/mL), medium quality control (MQC, 150 ng/mL), high quality control (HQC, 800 ng/mL). And 400 μL of de-protein solvent containing 200 ng/mL of tolbutamide (IS) was added in each sample, further processed identically to the *Sample preparation*.

**1.2 Method validation**

The developed LC-MS/MS method was validated mainly according to guidelines for Bioanalytical Method Validation published by the US Food and Drug Administration (FDA), with respect to specificity, linearity, accuracy, precision, matrix effect, recovery, stability under different conditions and dilution integrity.

*1.2.1 Specificity*

The specificity was validated through comparing the corresponding peaks of blank biological matrix to the blank matrix spiked with CAP, 5′-DFCR, 5′-DFUR and 5-FU and tolbutamide (IS) to exclude potential endogenous interference.

*1.2.2 Linearity*

The calibration curves of CAP, 5′-DFCR, 5′-DFUR and 5-FU were established by analyzing the cell lysates containing a standard over a range of 1–1000 ng/mL. These calibration curves were constructed by plotting the peak area ratios of the CAP, 5-FU, 5-DFUR, 5-DFCR to tolbutamide (IS) (y-axis) against the nominal concentration (x-axis) through weighed 1/x^2^ quadratic least-squares regression analysis at ten concentration levels (1–1000 ng/mL). LLOQ was defined as the lowest concentration of the calibration curve with an accuracy (relative error, RE, %) within ± 20% and precision (relative standard deviation, RSD, %) lower than 20%.

*1.2.3 Accuracy and precision*

Intra- and inter-day accuracy and precision of QC samples were assessed at four concentration levels of CAP, 5′-DFCR, 5′-DFUR and 5-FU in six replicates on one day and on three subsequent days: 1 ng/mL (LLOQ), 2.5 ng/mL (LQC), 150 ng/mL (MQC), and 800 ng/mL (HQC). Intra- and inter-day accuracy, the closeness to nominal value, was accepted within 15% for QC samples (except for LLOQ within ± 20%). Precision, the closeness of repeated individual measures, expressed as RSD of the same runs, should be ≤ 15% for QC samples and ≤ 20% for LLOQ.

*1.2.4 Matrix effect and extraction recovery*

Extraction recovery and matrix effects were determined at three concentration levels of CAP, 5′-DFCR, 5′-DFUR and 5-FU: 2.5 ng/mL (LQC), 150 ng/mL (MQC), and 800 ng/mL (HQC) in six independent samples. The biological matrix effect determined by comparing the analytical responses in extracted blank matrix samples spiked with corresponding concentrations of compounds, to that obtained from clean standard solutions at the same nominal concentration. The extraction recovery was calculated by comparing the analytical responses of extracted samples with those of post-extracted samples spiked with compounds. The results of the extraction recovery and matrix effect, expressed as RSD should be within ± 15%.

*1.2.5 Stability*

The stability of CAP, 5′-DFCR, 5′-DFUR and 5-FU (LQC 2.5 ng/mL, HQC 800 ng/mL) in stock solutions were investigated with six replicates under various storage conditions: 48 h at 4 ℃, short-term (room temperature, RT, 24 h), auto-sampler (4 ℃, 24 h), freeze-thaw (-80 ℃ to RT for 3 cycles), long-term (-80 ℃, 30 days). The stability results were calculated by comparing the analyzed with the nominal concentration and accepted when accuracy was within 15%.

*1.2.6 Dilution Integrity*

To confirm that dilution of cell lysate samples with high concentrations has no impact on the measured concentration, blank matrix was spiked with CAP, 5′-DFCR, 5′-DFUR and 5-FU at 600 or 800 ng/mL concentration and diluted 20 or 200 times with blank matrix. The accuracy should not vary more than ±15% and precision should be ≤15%.

**2. Result**

**2.1 Selectivity**

Ion transitions from parent ion to daughter ion of CAP, 5′-DFCR, 5′-DFUR, 5-FU and tolbutamide (IS) were shown in **Fig. S6**. Typical chromatograms of blank matrix, blank plasma spiked with CAP, 5′-DFCR, 5′-DFUR, 5-FU and tolbutamide (IS), and lysate sample of LoVo cells under co-cultured with HepG2 cells were illustrated in **Fig. S7**. The retention times for detecting CAP, 5′-DFCR, 5′-DFUR, 5-FU and tolbutamide (IS) were similar and the peaks were symmetrical. The method was selective and sensitive enough to enable efficient extraction, and the endogenous interference was not observed at the retention times.

**2.2 Linearity**

Representative standard curves for CAP, 5′-DFCR, 5′-DFUR and 5-FU were shown in **Fig. S8** and **Table S2**. The linearity for each curve was found to be greater than 0.99 using a weighted least squares linear regression method. The standard curve range was 1-1000 ng/mL and LLOQ was found to be 1 ng/mL for each compound.

**2.3 Accuracy and precision**

Data of intra- and inter-day accuracy and precision of CAP, 5′-DFCR, 5′-DFUR and 5-FU assessed with spiked cell lysate at 1 ng/mL (LLOQ), 2.5 ng/mL (LQC), 150 ng/mL (MQC), and 800 ng/mL (HQC) were summarized in **Table S3**. The intra- and inter-day accuracy values differed from -8.93% and 6.67% of nominal values, and the precision values were < 11.15% for all concentration levels, confirming that the method met the guideline’s requirements to quantify CAP, 5′-DFCR, 5′-DFUR and 5-FU in cell lysate.

**2.4 Extraction Recovery and Matrix Effects**

To ensure the quality of sample preparation, the extraction recovery and matrix effects were tested at three QC sample levels: 2.5 ng/mL (LQC), 150 ng/mL (MQC), and 800 ng/mL (HQC). The matrix effects of CAP, 5′-DFCR, 5′-DFUR and 5-FU were in the range of 98.12%-106.00% and all RSD were below 11.82%. The extraction recoveries were in the range of 98.75%-104.81% and all RSD were less than 10.29%. These results suggested that a sufficient recovery for all compounds was accepted and matrix effects could be excluded (**Table S4**).

**2.5 Stability**

The stability of CAP, 5′-DFCR, 5′-DFUR and 5-FU (LQC 2.5 ng/mL, HQC 800 ng/mL) under various storage conditions: 48 h at 4 ℃, short-term (room temperature, RT, 24 h), auto-sampler (4 ℃, 24 h), freeze-thaw (-80 ℃ to RT for 3 cycles), long-term (-80 ℃, 30 days). The stability results were calculated by comparing the analyzed with the nominal concentration and values of accuracy were within 15%. Good storage stability of CAP, 5′-DFCR, 5′-DFUR and 5-FU qualifies the method for routine use (**Table S5**).

**2.6 Dilution Integrity**

Examination of dilution integrity ensured that cell lysate samples out of the measurable range of an analyte could successfully be diluted to determine the original concentrations. Analysis of cell lysate spiked with CAP, 5′-DFCR, 5′-DFUR and 5-FU (600 or 800 ng/mL) and diluted 20- and 200-fold with blank cell lysate provided the required accuracy (**Table S6**).

**Fig. S6** Ion transitions from parent ion to daughter ion of CAP (**a**), 5′-DFCR (**b**), 5′-DFUR (**c**), 5-FU (**d**) and tolbutamide (IS) (**e**).


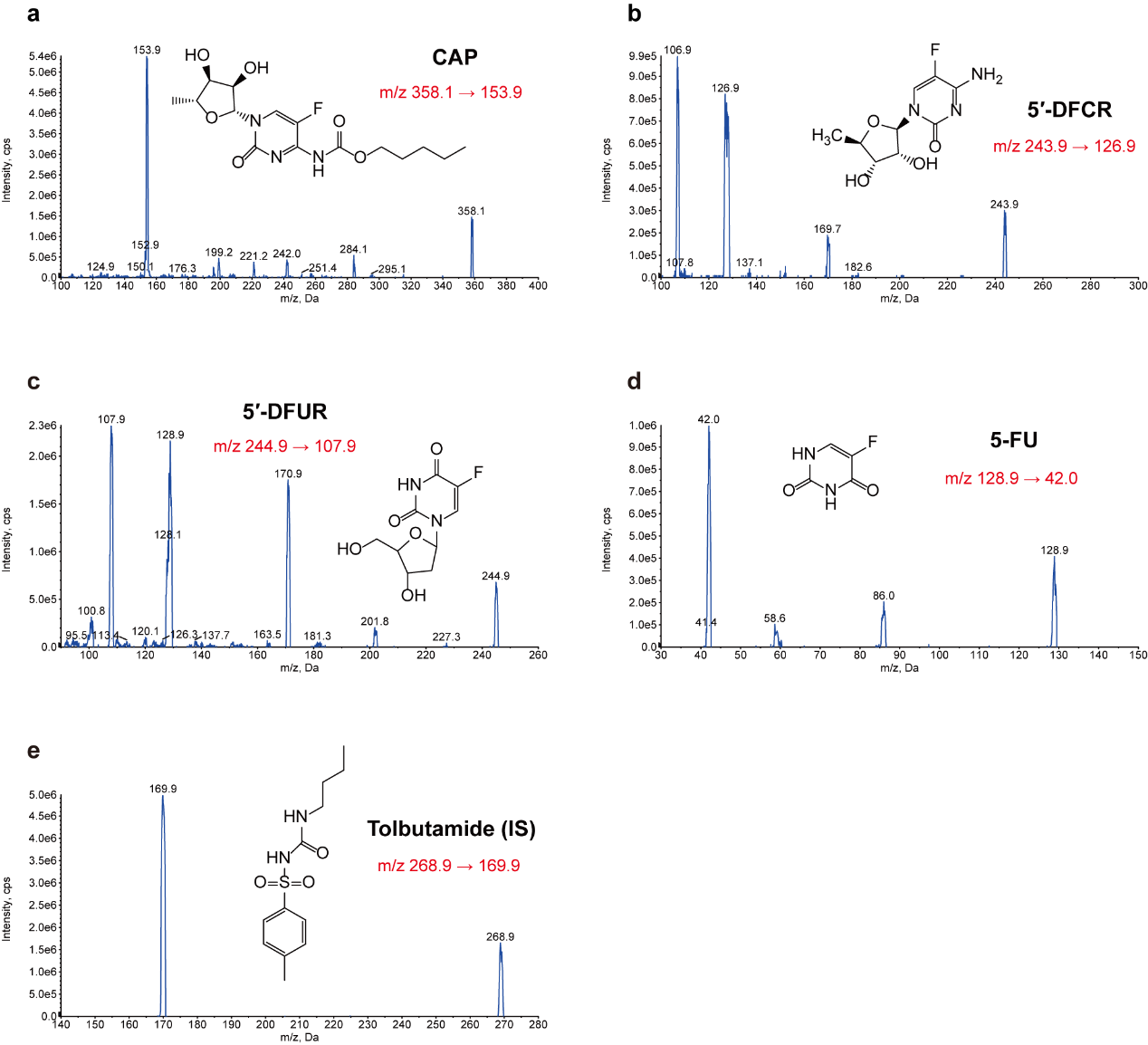


**Fig. S7** Representative chromatograms of CAP, 5′-DFCR, 5′-DFUR, 5-FU and tolbutamide (IS). **a** Blank matrix. **b**–**f** Blank matrix spiked with 150 ng/mL of CAP, 5′-DFCR, 5′-DFUR, 5-FU and 200 ng/mL of tolbutamide (IS). **g**–**k** CAP, 5′-DFCR, 5′-DFUR, 5-FU and tolbutamide (IS) in lysate sample of LoVo cells under co-cultured with HepG2 cells for 48 h.


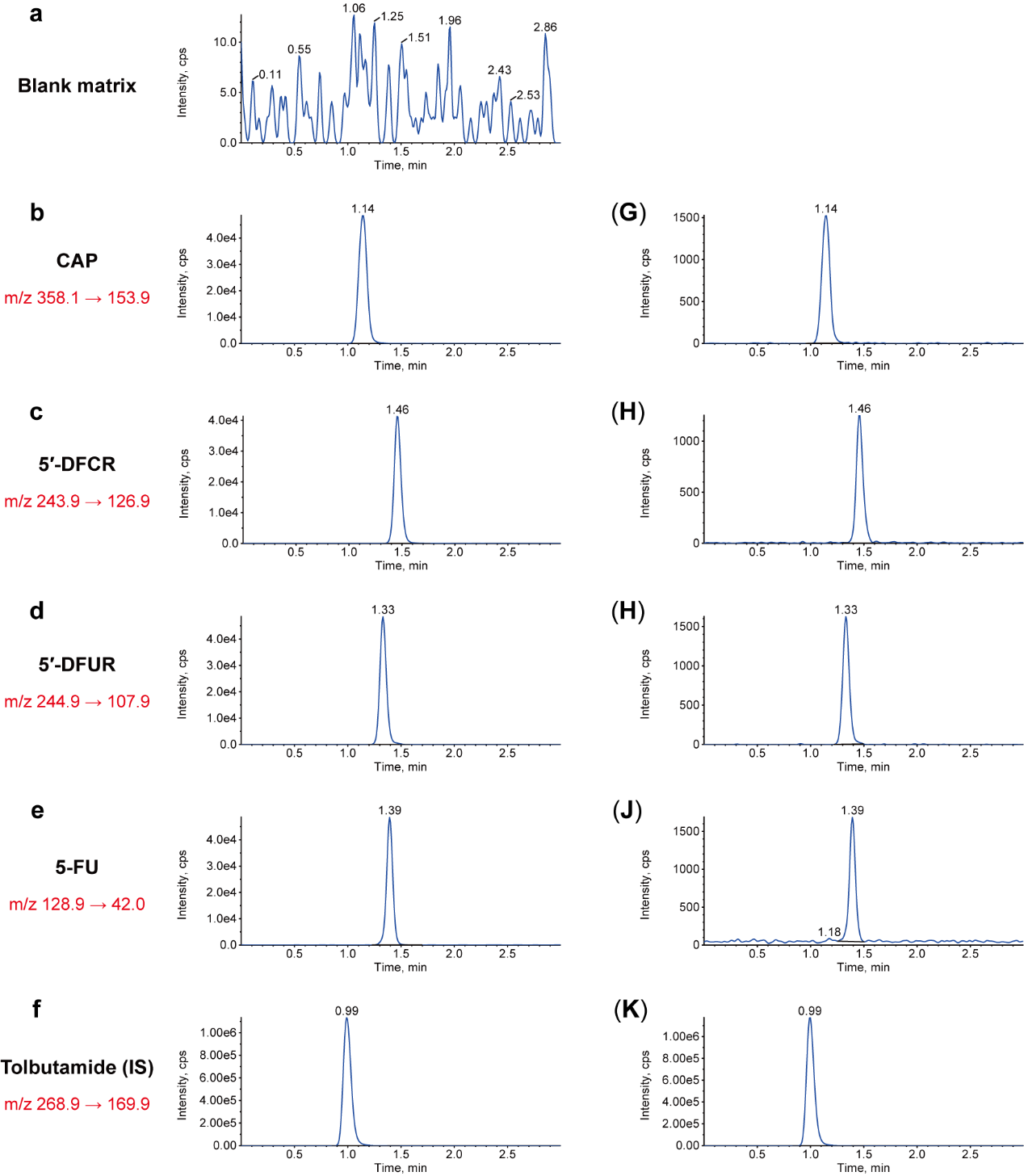


**Fig. S8** Calibration curves for CAP (**a**), 5′-DFCR (**b**), 5′-DFUR (**c**), 5-FU (**d**).


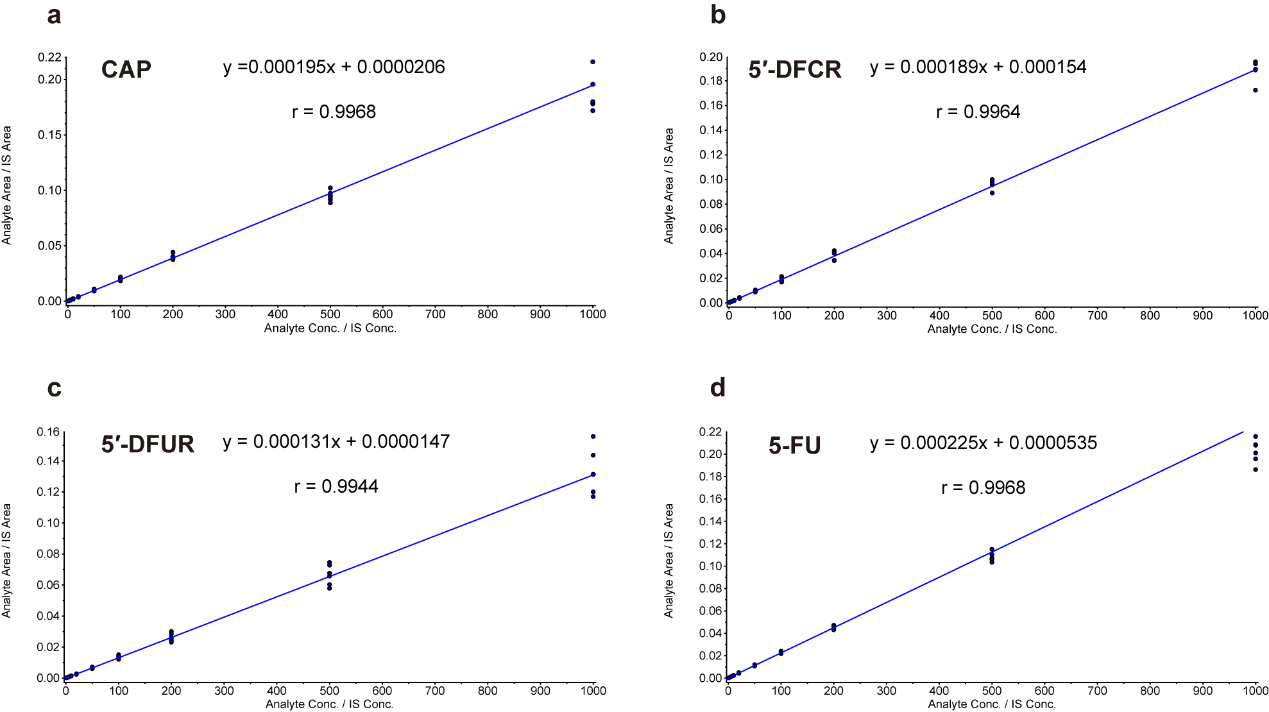


**Table S2** Calibration curves of CAP, 5′-DFCR, 5′-DFUR and 5-FU (n = 6).

| Analyte | Calibration curves | Correlation coefficients (r) | Linear ranges (ng/mL) |
| --- | --- | --- | --- |
| CAP | y =0.000195x + 0.0000206 | 0.9968 | 1-1000 |
| 5′-DFCR | y = 0.000189x + 0.000154 | 0.9964 | 1-1000 |
| 5′-DFUR | y = 0.000131x + 0.0000147 | 0.9944 | 1-1000 |
| 5-FU | y = 0.000225x + 0.0000535 | 0.9968 | 1-1000 |

**Table S3** Intra-assay and inter-assay accuracy and precision of CAP, 5′-DFCR, 5′-DFUR and 5-FU (n = 6).

| Analyte | Nominal conc. (ng/mL) | Intra-day | | |  | Inter-day | | |
| --- | --- | --- | --- | --- | --- | --- | --- | --- |
|  |  | Mean ± SD (ng/mL) | Accuracy  (RE, %) | Precision  (RSD, %) |  | Mean ± SD (ng/mL) | Accuracy  (RE, %) | Precision  (RSD, %) |
| CAP | 1 | 1.03 ± 0.10 | 2.98 | 9.56 |  | 1.02 ± 0.08 | 2.02 | 7.51 |
|  | 2.5 | 2.46 ± 0.15 | -1.73 | 6.06 |  | 2.55 ± 0.14 | 2.16 | 5.56 |
|  | 150 | 154.83 ± 2.71 | 3.22 | 1.75 |  | 157.00 ± 4.84 | 4.67 | 3.08 |
|  | 800 | 768.33 ± 14.11 | -3.96 | 1.84 |  | 789.00 ± 44.26 | -1.38 | 5.61 |
| 5′-DFCR | 1 | 0.91 ± 0.05 | -8.93 | 5.27 |  | 0.93 ± 0.05 | -7.48 | 5.67 |
|  | 2.5 | 2.54 ± 0.17 | 1.60 | 6.64 |  | 2.51 ± 0.15 | 0.22 | 5.81 |
|  | 150 | 159.50 ± 2.17 | 6.33 | 1.36 |  | 155.11 ± 5.11 | 3.41 | 3.29 |
|  | 800 | 798.00 ± 11.14 | -0.25 | 6.33 |  | 765.00 ± 27.93 | -4.38 | 3.65 |
| 5′-DFUR | 1 | 0.97 ± 0.05 | -3.15 | 5.38 |  | 0.96 ± 0.08 | -3.71 | 8.12 |
|  | 2.5 | 2.47 ± 0.16 | -1.13 | 6.62 |  | 2.54 ± 0.17 | 1.44 | 6.54 |
|  | 150 | 147.50 ± 3.56 | -1.67 | 2.42 |  | 152.61 ± 4.90 | 1.74 | 3.21 |
|  | 800 | 767.17 ± 6.68 | -4.10 | 0.87 |  | 784.61 ± 19.17 | -1.92 | 2.44 |
| 5-FU | 1 | 1.04 ± 0.12 | 4.03 | 11.15 |  | 1.02 ± 0.07 | 1.95 | 7.15 |
|  | 2.5 | 2.42 ± 0.15 | -3.33 | 6.22 |  | 2.58 ± 0.17 | 3.33 | 6.61 |
|  | 150 | 160.00 ± 2.53 | 6.67 | 1.58 |  | 153.94 ± 5.59 | 2.63 | 3.63 |
|  | 800 | 806.17 ± 9.75 | 0.77 | 1.21 |  | 743.50 ± 47.34 | -7.06 | 6.37 |

RE, relative error; RSD, relative standard deviation.

**Table S4** Assessment of the matrix effect and recovery of CAP, 5′-DFCR, 5′-DFUR and 5-FU (n = 6).

| Analyte | Nominal conc. (ng/mL) | Matrix effect | |  | Recovery | |
| --- | --- | --- | --- | --- | --- | --- |
|  |  | Mean ± SD (%) | RSD (%) |  | Mean ± SD (%) | RSD (%) |
| CAP | 2.5 | 106.00 ± 6.86 | 6.47 |  | 100.45 ± 7.63 | 7.59 |
|  | 150 | 104.84 ± 2.51 | 2.39 |  | 102.10 ± 3.65 | 3.57 |
|  | 800 | 100.99 ± 3.21 | 3.23 |  | 104.11 ± 2.94 | 2.83 |
| 5′-DFCR | 2.5 | 104.35 ± 12.34 | 11.82 |  | 100.21 ± 9.59 | 9.57 |
|  | 150 | 102.96 ± 2.45 | 2.37 |  | 98.75 ± 3.12 | 3.16 |
|  | 800 | 98.73 ± 3.43 | 3.47 |  | 101.36 ± 2.24 | 2.21 |
| 5′-DFUR | 2.5 | 99.81 ± 5.82 | 5.83 |  | 104.81 ± 9.22 | 8.80 |
|  | 150 | 102.89 ± 2.88 | 2.80 |  | 102.43 ± 3.28 | 3.20 |
|  | 800 | 101.85 ± 2.10 | 2.06 |  | 102.71 ± 2.55 | 2.48 |
| 5-FU | 2.5 | 102.01 ± 7.29 | 7.15 |  | 101.48 ± 10.44 | 10.29 |
|  | 150 | 103.45 ± 1.99 | 1.92 |  | 101.94 ± 2.48 | 2.43 |
|  | 800 | 98.12 ± 3.20 | 3.26 |  | 103.20 ± 3.78 | 3.66 |

RSD, relative standard deviation.

**Table S5** Stability results of CAP, 5′-DFCR, 5′-DFUR and 5-FU (n = 3).

| Analyte | Stability test | Nominal conc. (ng/mL) | Mean ± SD | RE (%) | RSD (%) |
| --- | --- | --- | --- | --- | --- |
| CAP | 4 ℃, 48 h | 2.5 | 2.67 ± 0.08 | 6.80 | 3.07 |
|  |  | 800 | 856.00 ± 12.12 | 7.00 | 1.42 |
|  | Short-term  (RT, 24 h) | 2.5 | 2.69 ± 0.05 | 7.60 | 1.97 |
|  |  | 800 | 844.67 ± 4.04 | 5.58 | 0.48 |
|  | Auto-sampler  (4 ℃, 24 h) | 2.5 | 2.67 ± 0.05 | 6.93 | 1.77 |
|  |  | 800 | 824.00 ± 8.66 | 3.00 | 1.05 |
|  | Freeze-thaw  (-80 ℃ to RT for 3 cycles) | 2.5 | 2.69 ± 0.05 | 7.47 | 1.87 |
|  |  | 800 | 869.33 ± 17.04 | 8.67 | 1.96 |
|  | Long-term  (-80 ℃, 30 days) | 2.5 | 2.68 ± 0.07 | 7.20 | 2.59 |
|  |  | 800 | 851.33 ± 13.05 | 6.42 | 1.53 |
| 5′-DFCR | 4 ℃, 48 h | 2.5 | 2.55 ± 0.22 | 2.13 | 8.42 |
|  |  | 800 | 780.33 ± 7.23 | -2.46 | 0.93 |
|  | Short-term  (RT, 24 h) | 2.5 | 2.56 ± 0.14 | 2.27 | 5.31 |
|  |  | 800 | 780.33 ± 23.12 | -2.46 | 2.96 |
|  | Auto-sampler  (4 ℃, 24 h) | 2.5 | 2.61 ± 0.05 | 4.27 | 1.77 |
|  |  | 800 | 781.33 ± 22.94 | -2.33 | 2.94 |
|  | Freeze-thaw  (-80 ℃ to RT for 3 cycles) | 2.5 | 2.58 ± 0.13 | 3.33 | 4.87 |
|  |  | 800 | 805.00 ± 27.00 | 0.63 | 3.35 |
|  | Long-term  (-80 ℃, 30 days) | 2.5 | 2.69 ± 0.09 | 7.60 | 3.35 |
|  |  | 800 | 783.67 ± 16.17 | -2.04 | 2.06 |
| 5′-DFUR | 4 ℃, 48 h | 2.5 | 2.72 ± 0.11 | 8.67 | 4.05 |
|  |  | 800 | 833.67 ± 18.01 | 4.21 | 2.16 |
|  | Short-term  (RT, 24 h) | 2.5 | 2.78 ± 0.06 | 11.07 | 2.32 |
|  |  | 800 | 827.67 ± 8.14 | 3.46 | 0.98 |
|  | Auto-sampler  (4 ℃, 24 h) | 2.5 | 2.73 ± 0.02 | 9.33 | 0.56 |
|  |  | 800 | 839.33 ± 24.54 | 4.92 | 2.92 |
|  | Freeze-thaw  (-80 ℃ to RT for 3 cycles) | 2.5 | 2.75 ± 0.06 | 10.13 | 2.31 |
|  |  | 800 | 846.00 ± 10.44 | 5.75 | 1.23 |
|  | Long-term  (-80 ℃, 30 days) | 2.5 | 2.68 ± 0.08 | 7.07 | 3.00 |
|  |  | 800 | 843.00 ± 22.52 | 5.38 | 2.67 |
| 5-FU | 4 ℃, 48 h | 2.5 | 2.62 ± 0.08 | 4.67 | 3.18 |
|  |  | 800 | 697.67 ± 16.80 | -12.79 | 2.41 |
|  | Short-term  (RT, 24 h) | 2.5 | 2.50 ± 0.15 | 0.00 | 5.97 |
|  |  | 800 | 707.00 ± 14.73 | -11.63 | 2.08 |
|  | Auto-sampler  (4 ℃, 24 h) | 2.5 | 2.57 ± 0.13 | 2.67 | 5.19 |
|  |  | 800 | 752.00 ± 11.36 | -6.00 | 1.51 |
|  | Freeze-thaw  (-80 ℃ to RT for 3 cycles) | 2.5 | 2.62 ± 0.04 | 4.80 | 1.38 |
|  |  | 800 | 741.67 ± 74.07 | -7.29 | 9.99 |
|  | Long-term  (-80 ℃, 30 days) | 2.5 | 2.66 ± 0.10 | 6.40 | 3.92 |
|  |  | 800 | 710.67 ± 11.02 | -11.17 | 1.55 |

RT, room temperature; RE, relative error; RSD, relative standard deviation.

**Table S6** Dilution integrity of CAP, 5′-DFCR, 5′-DFUR and 5-FU (n = 6).

| Analyte | Concentration  Spiked (ng/mL) | Dilution factor | Mean conc. (ng/mL) | Accuracy  (RE, %) | Precision  (RSD, %) |
| --- | --- | --- | --- | --- | --- |
| CAP | 600 | 200 | 604.67 ± 21.63 | 0.78 | 3.58 |
|  | 800 |  | 820.67 ±24.68 | 2.58 | 3.01 |
|  | 600 | 20 | 596.50 ± 13.79 | -0.58 | 2.31 |
|  | 800 |  | 799.50 ± 17.35 | -0.06 | 2.17 |
| 5′-DFCR | 600 | 200 | 660.17 ± 12.46 | 10.03 | 1.89 |
|  | 800 |  | 863.67 ± 23.06 | 7.96 | 2.67 |
|  | 600 | 20 | 656.17 ± 14.63 | 9.36 | 2.23 |
|  | 800 |  | 889.83 ± 9.11 | 11.23 | 1.02 |
| 5′-DFUR | 600 | 200 | 628.33 ± 27.32 | 1.98 | 4.35 |
|  | 800 |  | 823.67 ± 35.30 | 1.04 | 4.29 |
|  | 600 | 20 | 626.00 ± 12.39 | 4.33 | 4.72 |
|  | 800 |  | 837.50 ± 8.71 | 4.69 | 2.96 |
| 5-FU | 600 | 200 | 657.33 ± 24.16 | 9.56 | 3.68 |
|  | 800 |  | 838.00 ± 35.89 | 4.75 | 4.28 |
|  | 600 | 20 | 623.83 ± 15.07 | 3.97 | 2.41 |
|  | 800 |  | 816.00 ± 19.49 | 2.00 | 2.39 |

RE, relative error; RSD, relative standard deviation.
